# Zn salts incorporated polyurethane/polyacrylonitrile electrospinning fiber membrane for high porosity polymer electrolyte in Zn ion battery

**DOI:** 10.1038/s41598-023-43962-6

**Published:** 2023-10-05

**Authors:** Chutiwat Likitaporn, Manunya Okhawilai, Nangan Senthilkumar, Tawan Wongsalam, Nattapon Tanalue, Pornnapa Kasemsiri, Jiaqian Qin, Hiroshi Uyama

**Affiliations:** 1 Nanoscience and Technology Interdisciplinary Program, Graduate School, Chulalongkorn University, Bangkok, 10330 Thailand; 2 Metallurgy and Materials Science Research Institute, Chulalongkorn University, Bangkok, 10330 Thailand; 3 Center of Excellence in Responsive Wearable Materials, Chulalongkorn University, Bangkok, 10330 Thailand; 4 Multidisciplinary Program in Petrochemistry and Polymer Science, Faculty of Science, Chulalongkorn University, Bangkok, 10330 Thailand; 5 Sustainable Infrastructure Research and Development Center and Department of Chemical Engineering, Faculty of Engineering, Khon Kaen University, Khon Kaen, 40002 Thailand; 6 Department of Applied Chemistry, Graduate School of Engineering, Osaka University, Osaka, 565-0871 Japan

**Keywords:** Batteries, Batteries, Polymer characterization

## Abstract

So far, a large variety of polymer molecule architectures have been explored in the electrolyte field. Polymer electrolytes have gathered research efforts as an interesting alternative to conventional liquid electrolytes due to their advantages of low probability of leakage and low volatility of liquid solvent, lightweight, flexibility, inertness, high durability, and thermal stability. In this work, a polymer electrolyte developed from a polyurethane/polyacrylonitrile (PU/PAN) electrospinning fiber membrane was added with different zinc (Zn) salts, namely, Zn(CH_3_CO_2_)_2_, ZnSO_4_, and Zn(OTf)_2_. The samples with the Zn salt presented many different properties; especially, the high Zn(OTf)_2_ sample showed gradually bundle morphology in its structure. Characterization revealed improved properties in contact angle, water uptake, and thermal resistance. Namely, the 15 wt% Zn(OTf)_2_) sample exhibited an outstandingly high ionic conductivity of 3.671 mS cm^−1^, which is 10 times higher than that of the neat PU/PAN membrane.

## Introduction

Extremely concerned topics in the present years are the rise in energy demand^1^, electric vehicles (EVs)^2^, environmental sustainability^3^ and environmental pollution^4^, which help accelerate the development of renewable energy sources and related energy storage technologies^5^. Energy storage systems play an important role in figuring out the unstable energy supply from renewable sources. In addition, emerging advancements in portable and wearable electronic devices are a driving force in developing high-performance and high-safety batteries for energy storage.

Recently, Zinc ion batteries (ZIBs) and Sodium ion batteries (SIBs) have been used as better alternatives to Lithium-ion batteries (LIBs)^6,7^. The theoretical capacity of ZIBs (820 mAh g^-1^) and SIBs (1165 mAh g^-1^) is comparatively higher than that of LIBs (200–450 mAh g^-1^), making great attention toward ZIBs and SIBs exploration^7–10^. However, the low rate capability and lower energy density, short cycling stability, and dependency of complex electrolytes in SIBs create a barrier to their wide utility. On the other side, the ZIBs have attracted significant interest due to their safety, environmental friendliness, and use of non-explosive and low-toxic electrolytes. Furthermore, distinct advantages of metallic Zn, namely, its large theoretical capacity (820 mAh g^-1^ and 5855 mAh cm^-3^)^11^, low standard redox potential (− 0.76 V vs. SHE)^12^, high abundance (approximately 300 times higher than that of Li)^13^, and low cost (~ 20 times lower than that of Li)^14,15^, have encouraged the development of ZIBs. However, the major challenges in normal ZIBs may obstruct the wide spread of these technologies, such as the poor electrochemical reversibility in aqueous electrolyte, dendrite growth, and zinc passivation on the electrode^16^. The intrinsic issues of Zn metal anodes, namely, hydrogen evolution, dendrite growth, and electrode passivation, have prevented their implementation in commercial rechargeable batteries until now.

An effective polymer electrolyte has a high ionic conductivity, prevents electrode contact, presents appropriate mechanical properties, and, especially, be an ion transport medium affecting further electrochemical properties in battery cells. Ion-conducting polymers coordinate with alkali-metal salts through the direct interaction of the metal cation with an unbound electron pair. To enable ion transport in the polymer matrix, the polymer structure should have polar groups with electron donor groups to affect interaction with the salt^17^. Furthermore, these properties are also essential to the application of polymer electrolytes: safety-concerned properties such as mechanical properties, thermal resistance, and physical properties^18^. Due to these significant characteristics, several polymer materials have been continually developed as functional materials in energy technologies, including fuel cells^19–21^ and metal-ion batteries^6,22^. Polymer electrolytes can be classified based on material composition: solid polymer electrolyte (SPE)^23^, plasticized polymer electrolyte^24^, gel polymer electrolyte (GPE)^25^, and composite polymer electrolyte (CPE)^26^.

The development of polymer electrolytes has become one of the most effective ways to enhance battery performances. Polyacrylonitrile (PAN) has been evaluated as a polymer electrolyte. The interaction between the acrylonitrile (−CN) group and the Li ion was investigated to supports its application as a polymer electrolyte^27^. Recently, Bao et al. proposed a novel polymer electrolyte developed from polycarbonate-based polyurethane (PCPU) containing the polar carbonated group (−COO) resulting in high ionic conductivity in a Li battery^28^. However, this PCPU electrolyte proposed for Li batteries still exhibited ion conductivity at quite a high temperature (80 °C). Another group applied an electrospinning-prepared GPE from three polymer components including poly(vinylidene fluoride) (PVDF), thermoplastic polyurethane, and PAN to produce a high-porosity membrane for use in Li-ion batteries^29^. This GPE with high electrolyte uptake was able to provide high ionic conductivity even at ambient temperature. However, costly materials, for example PVDF, may limit its economical scale-up. Electrospun fiber mats can be prepared to provide these required characteristics. In addition, a high-stability lithium metal battery with GPE based on poly(vinylidene fluoride hexafluoropropylene) (PVDF-HFP) incorporated BaTiO_3_ was developed by using the electrospinning technique^30^. The spinning solution can be prepared from various polymer matrixes that contain suitable functional groups for ion interaction, and a nano-inorganic filler can be incorporating into the electrospun fiber^31^. Nano- to micro-sized electrospinning fibers show specific surface behavior for electrolyte and electrode interaction.

For ZIB electrolytes, the polymer electrolyte or GPE includes Zn salt as an ionic medium in a battery cell. The ion transport mechanism is promoted by using these ionic salts. For instance, Zn(CH_3_CO_2_)_2_ salt has been added to polyethylene glycol (PEG)-based electrolytes, resulting in a high ionic conductivity of 1.55 × 10^−6^ S cm^−1^, obtained from an optimum ratio of polymer to salt^18^. Recently, different types of zinc salts such as Zn(CH_3_CO_2_)_2_, Zn(OTf)_2_, and ZnSO_4_ have been studied as additives to the GPE^21,32^. The GPEs modified with different zinc salts have affected the entire electrochemical properties of the electrochemical cell^21^. In addition, the development of solid electrolytes from porous materials was able to decrease the crystallinity structure and improve electrochemical properties^33^.

This study focuses on developing a Zn salt-incorporated high-porous polymer electrolyte membrane from PU/PAN with improved properties. Here, the PU was selected due to the flexibility in their structure, besides, the urethane functional group (–COONH) can coordinate with positive ions^34^, making it have better binding ability with Zn^2+^. However, due to its lower molecular weight, PU has not produced electrospun nanofibers (Fig. S1). In the case of PAN, the absence of positive ion binding ability of PAN, it cannot used for electrolyte preparation. However, its high mechanical property helps to prepare stable fiber formation. Based on these insights, the optimum mixing of PU and PAN has been used for electrospun fiber-based electrolyte production^35^. The fiber membrane was synthesized by electrospinning, an effective fiber preparation method, to prepare a salt-attached porous fiber membrane. Furthermore, its physical, mechanical, thermal, and electrochemical properties were studied for use as a polymer electrolyte.

## Materials and methods

### Materials

PAN (average Mw of 150,000 g mol^−1^), dibutyltin dilaurate (95%), Zn(OTf)_2_ (98%), Zn(CH_3_CO_2_)_2_, and ZnSO_4_ were purchased from Sigma Aldrich (USA). Poly(hexamethylene) carbonate diol (average Mn of 2000 g mol^−1^), UH200, was supplied from UBE Chemicals (Thailand). 1,4-Butanediol (AR grade) was purchased from QReC (New Zealand). Dimethylformamide (DMF) was supplied by RCI Labscan Ltd. (Thailand). A partially bio-based diisocyanate (Tolonate X FLO 100) was provided by Vencorex Co. Ltd. (France).

### Synthesis of bio-based PU

PU was synthesized following a previously published method^36^. In a four-necked round-bottom flask equipped with a mechanical stirrer, thermometer, and condenser, polycarbonate diol, partially bio-based diisocyanate, and 1,4-butanediol were mixed at a molar ratio of 1:3:2 in DMF, which was added as the reaction solvent. The reaction was carried out for 6 h under nitrogen at 70 °C. Afterward, the resulting product was purified with ethanol and dried before further use.

### Electrospinning of PAN/bio-based PU

**Figure 1:**
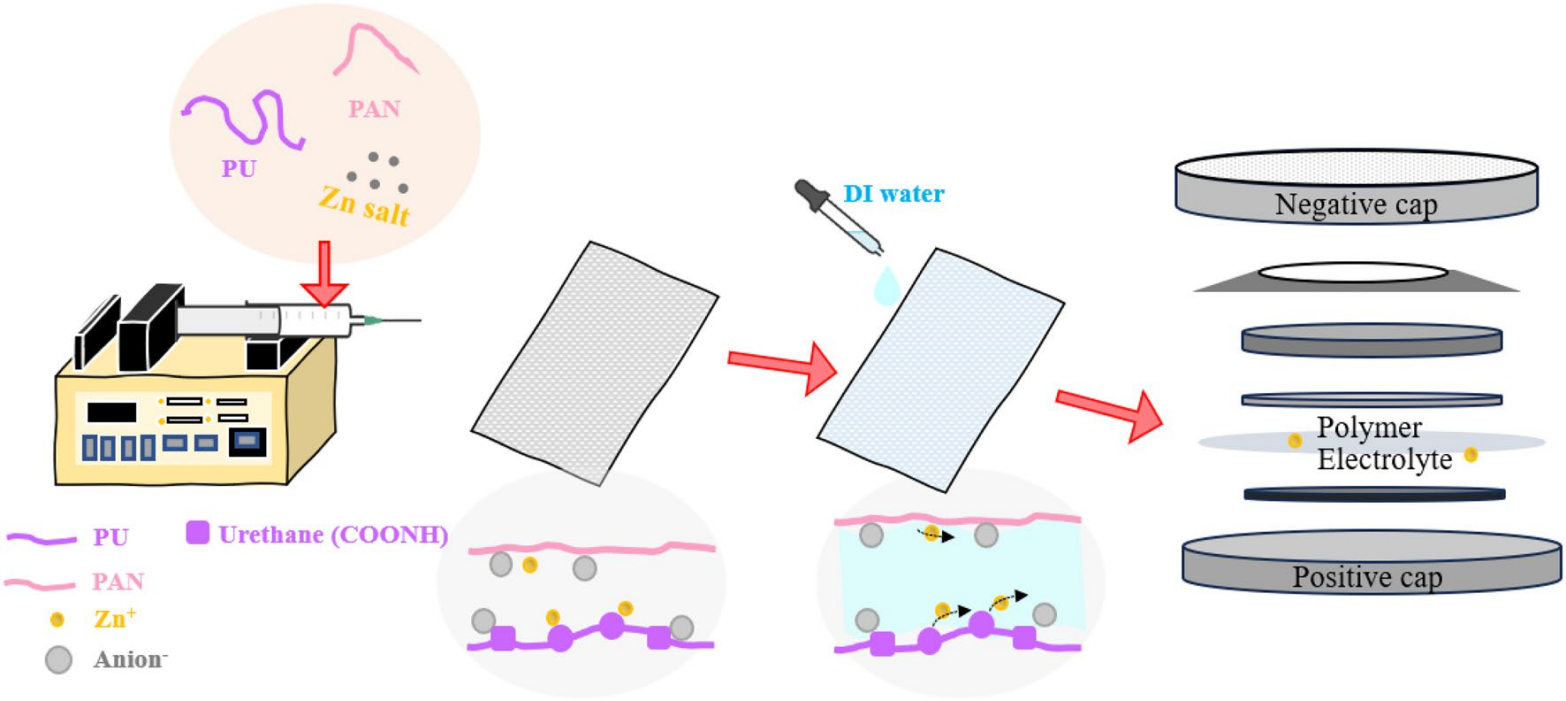
Schematic illustration of PU/PAN/ZnX.

PU/PAN electrospun fiber mats with a blending weight ratio of 50:50 were prepared by electrospinning. As illustrated in Figs. 1 and S2, the equipment consisted of a high-voltage power supply, volumetric syringe pump, and fiber collector, which were supplied by IBA Company (Thailand). The PU/PAN solution with a weight ratio of 50/50 and 12 wt% was dissolved in DMF via mechanical stirring, and different zinc salts, including Zn(CH_3_CO_2_)_2_, Zn(OTf)_2_, and ZnSO_4_, were added to the PU/PAN solution with a weight fraction of 0–15 wt%. The solution with zinc salt was stirred overnight until a homogeneous solution was obtained. The suitable blending weight ratio of PU/PAN was then fabricated into non-woven fiber mats. The homogeneous PU/PAN solution was charged into a 5 mL syringe coupled with a needle with an inner diameter of 0.55 mm and placed in a syringe pump with a controlled flow rate of the polymer solution of 1.0 mL/h at a voltage of 15 kV and a needle tip to collector distance of 19 cm. The parameter conditions provide quite fine, perfect fiber morphology for every membrane. Humidity and temperature during the electrospinning were controlled using an environmental chamber, 50% ± 10% and 25 °C ± 2 °C, respectively. The electrospun fiber mats were dried for at least 24 h before further use. The mats are represented as PU/PAN/ZnX-y, where X is the type of Zn salt [A = Zn(CH_3_CO_2_)_2_, S = ZnSO_4_, or T = Zn(OTf)_2_] and y is the amount of zinc salt in weight percentage.

### Characterization

The morphology of PU/PAN/ZnX-y electrospun fiber mats was determined by scanning electron microscopy (SEM) (Hitachi SU-4800, Japan) equipped with energy dispersive X-ray spectroscopy at a voltage of 3.0 kV and an emission current of 10 mA. The surfaces of the samples were sputter-coated with gold before measurement. The average fiber diameter in all SEM images was measured with ImageJ.

The chemical structures of PU/PAN/ZnX-y were investigated using Fourier-transform infrared spectroscopy (FT-IR; Bruker FT-IR Alpha II, United State) equipped with an attenuated total reflectance accessory. All samples were scanned at a resolution of 4 cm^−1^ within the spectral range of 4000–500 cm^−1^. The obtained results were subtracted from the background spectra.

The mechanical behavior of PU/PAN/ZnX-y was tested at room temperature with a universal testing machine (LLOYD model LF Plus). The specimens with a dimension of 10 mm × 100 mm × 0.02 mm (length × width × depth) were prepared. Gauge length and cross-head speed were 50 mm and 4 mm min^−1^, respectively.

The thermal transitions of specimens were investigated using differential scanning calorimetry (DSC; Netzch DSC 3500 Sirius, German). The sample was contained in an aluminum pan, while the reference was determined as empty aluminum pan. The testing condition was set in the range of −100 to 220 °C at a heating rate of 10 °C min^−1^ under nitrogen.

The thermal degradation and char yield of PU/PAN and zinc salt-incorporated samples were studied using thermo gravimetric analyzer (TGA, Shimadzu). The temperature was operated from room temperature to 700 °C at 20 °C min^−1^ heating rate in a nitrogen atmosphere.

The dimensional stability of fiber samples was investigated using a modified method from Saisangtham et al.^37^. The fiber was prepared in a circular shape with 19 mm of diameter and then heated in an oven at a temperature of 80, 100, and 120 °C for 1 h each.

The electrospun fiber mat samples were cut into 2 × 2 cm^2^ squares. The electrolyte uptake of the samples was determined using Eq. (1).

1 $$\mathrm{Electrolyte\, uptake }\left(\mathrm{\%}\right)= \frac{{\mathrm{W}}_{\mathrm{w}}-{\mathrm{W}}_{\mathrm{d}}}{{\mathrm{W}}_{\mathrm{d}}}\times 100$$

where ${W}_{d}$ and ${W}_{w}$ are the weights of the PU/PAN/ZnX-y electrospun fiber mats before and after immersing in deionized water for 24 h, respectively. The average was calculated from three measurements.

The wettability of the fiber mats by an electrolyte was observed with electrolyte droplets placed on their surfaces and confirmed using an in-house contact angle analyzer. The electrospun fiber mats were dried in a vacuum overnight to remove all moisture. Contact angle measurements were conducted within 5 s by placing one drop of DI water on the samples. The final contact angles were obtained as the average of three measurements at room temperature.

A potentiostat/galvanostat (PSTrace4 PalmSens, The Netherlands) was utilized for investigating the electrochemical properties. The measurements were performed on an applied 10 mV AC potential from 1 MHz to 1 Hz. The SS/Polymer electrolyte/SS cell was constructed by inserting the fiber mats between stainless steel blocking electrodes. The membrane thickness and active area were approximately 100–300 $\upmu$m and 1.54 cm^2^, respectively. Transference number measurement was performed by the DC polarization method using chronoamperometry. A polarization voltage of 10 mV was applied across the sample, and the initial maximum current *I*_0_ and steady-state current *I*_s_ were recorded. Equation (2) was used to calculate ionic conductivity (δ). The average value was from three measurements.

2 $$\delta (s /cm)=\frac{\mathrm{d}}{{\mathrm{R}}_{b}\mathrm{ x S}}$$

where d refers to the thickness of the fiber membrane, R_b_ is the bulk ionic resistance of the membrane, which can be obtained from the plot, and S is the area of electrodes connected within the membrane. The Zn^2+^ transference number (t^+^) was calculated by the Bruce-Vincent method using Eq. (3)^38,39^:

3 $${t}^{+}=\frac{{I}_{s}(\Delta V-{I}_{0}{R}_{0})}{{I}_{0}(\Delta V-{I}_{s}{R}_{s})}$$

where ∆V represents the testing step potential; I_0_ and I_s_ are the currents at the initial and steady states, respectively; and R_0_ and R_S_ represent the cell resistance before and after polarization, respectively.

The dissolution of zinc salt in a highly porous polymer electrolyte was evaluated by immersing the membrane in 20 ml of DI water for 24 h. Then, the obtained solutions were brought to test by inductively coupled plasma (ICP-OES, Shimadzu, ICPE900, Kyoto, Japan) and the concentration of zinc in water was calculated in parts per million (ppm).

The voltage response on the electrochemical compatibility of PU/PAN/ZnX-y fiber mats was investigated and recorded as a function of cycle at room temperature for long-term zinc charge/discharge cycles using a Neware testing system (Shenzhen Neware CT-4008, China). The charge–discharge cycles of the CR2032 coin cell with symmetric Zn/polymer electrolyte/Zn cells and adding a drop of DI water were performed to get quasi-solid polymer electrolyte at a current density of 0.25 mA cm^−2^. The CR2032 coin cells were assembled by sandwiching the polymer electrolyte between Zn foil and a VNO electrode in deionized water to measure the potential range. The cathode NVO fabrication is explained in the supplementary file. Then, the coin cells equipped with Zn/PU/PAN/NVO and Zn/PU/PAN/ZnT-15/NVO adding a drop of 0.5 M Zn(OTf)_2_ solution were brought for rate performance testing. The setting conditions for different current densities were 0.1, 0.2, 0.4, 0.6, 0.8, 1.0, 2.0, and 5.0 A g^-1^, then returned to 0.2 A g^−1^.

## Results and discussion

### Morphology of the Zn salt incorporated PAN/PU electrospun fiber mats with different types of Zn salts and ratios

The electrospun membrane was prepared by electrospinning at 1 mL h^−1^ polymer solution feeding rate, 15 kV applied voltage, and 19 cm distance from the needle tip to the collector. The morphologies of the PAN/PU electrospun membranes with different Zn salts, i.e., Zn(OTf)_2_, (Zn(CH_3_CO_2_)_2_), or (ZnSO_4_), at 0–15 wt% were characterized by SEM. Shear force, which drives the polymer solution at the needle wall, was fixed at a constant rate.

**Figure 2:**
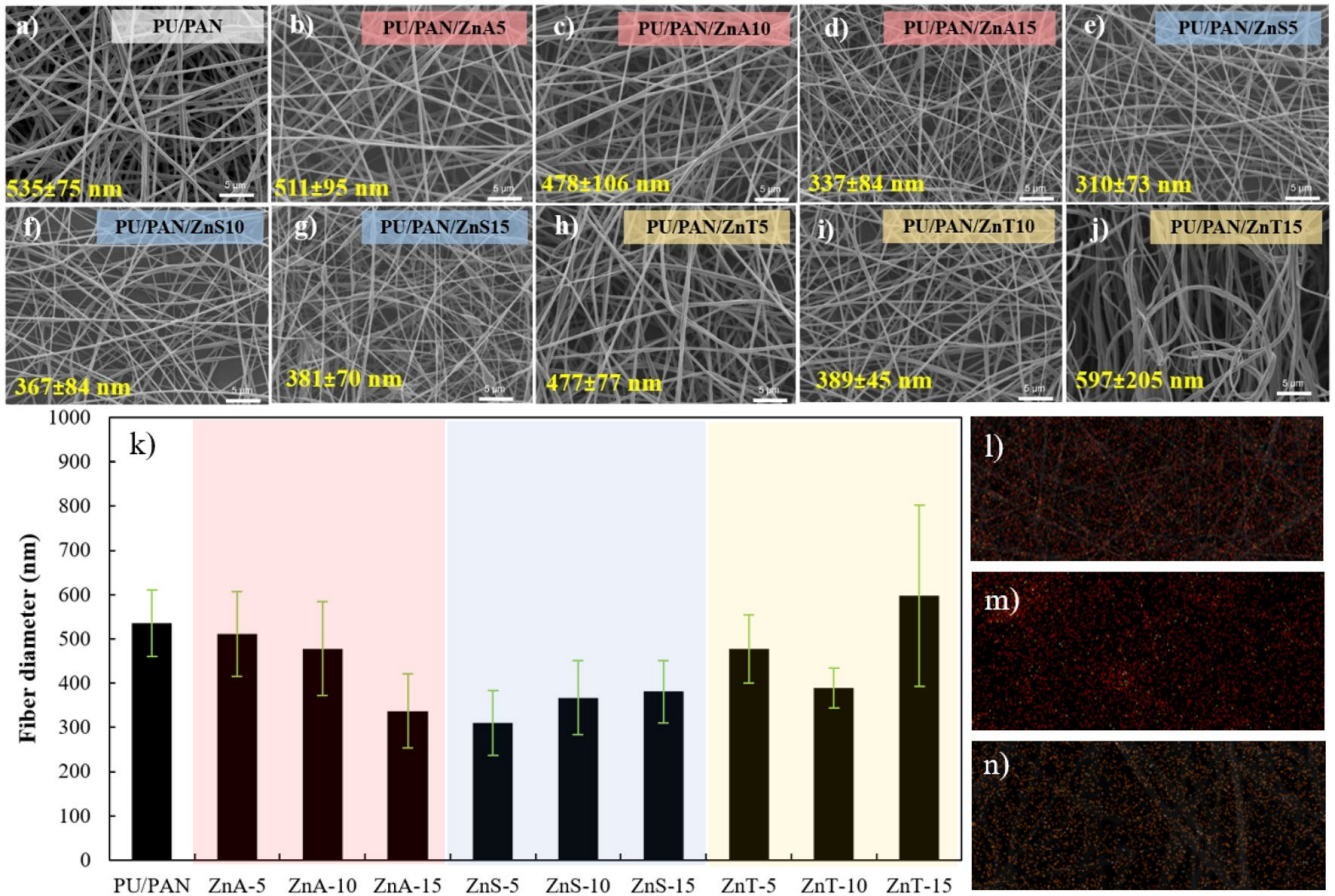
SEM micrograph of (**a**) PU/PAN (**b**) PU/PAN/ZnA-5, (**c**) PU/PAN/ZnA-10, (**d**) PU/PAN/ZnA-15, (**e**) PU/PAN/ZnS-5, (**f**) PU/PAN/ZnS-10, (**g**) PU/PAN/ZnS-15, (**h**) PU/PAN/ZnT-5, (**i**) PU/PAN/ZnT-10, (**j**) PU/PAN/ZnT-15, and (**k**) plot of fiber diameter of PU/PAN/ZnX-y, and Zn elemental mapping of (**l**) PU/PAN/ZnA-15, (**m**) PU/PAN/ZnS-15, and (**n**) PU/PAN/ZnT-15.

Figure 2 shows the morphologies and fiber diameters of all Zn-incorporated PU/PAN electrospun membranes. The pure PU/PAN membrane without Zn salt reveals a smooth and random fiber morphology with an average fiber diameter of 535 ± 75 nm. The PU/PAN/ZnA-y fiber mats presented a slight decrease in fiber diameter compared to the neat PU/PAN and a decrease in fiber content with increasing Zn(CH_3_CO_2_)_2_ content. In Fig. 2a–d, the fiber diameters of PU/PAN/ZnA-5, PU/PAN/ZnA-10, and PU/PAN/ZnA-15 were 511 ± 95, 478 ± 106, and 337 ± 84 nm, respectively. The decrease in fiber diameter is due fiber extension morphology from the addition of the salt molecule in the polymer solution. Next, ZnSO_4_ salt was incorporated to synthesize PU/PAN/ZnS-5, PU/PAN/ZnS-10, and PU/PAN/ZnS-15, as shown in Fig. 2e–g. The fiber diameter decreased in PU/PAN/ZnS. However, the average fiber diameter was not clearly decreased by the addition of ZnSO_4_ salt. The fiber diameter of ZnS-5 was decreased to 310 ± 73 nm compared with that of the non-salt fiber, and the fiber diameter appeared stable with measured sizes of 367 ± 84 and 381 ± 70 nm as the ZnSO_4_ salt contents increased to 10 and 15wt%, respectively. This turnaround in trend toward increasing fiber diameter from salt addition may be due to the high amount of salt in polymer fiber and fiber connections. Moreover, the fiber morphologies of PUPAN with Zn(OTf)_2_ at ratios of ZnT-5, ZnT-10, and ZnT-15 are exhibited in Fig. 2h–j. The ZnT samples exhibited decreasing fiber diameter, a similar trend to Zn(CH_3_CO_2_)_2_ salt-included samples in 5% and 10% salt ratios, but ZnT-3 exhibited increasing fiber diameter with a measured size of 597 ± 205 nm. The size distribution of all electrospun fiber mats is illustrated in Table S1. The comparison of average fiber diameter is clearly presented in Fig. 2k. Besides, the zinc element mappings were investigated to confirm the presence of zinc from zinc salt by SEM-equipped energy dispersive X-ray spectroscopy (EDS) and presented as in Fig. 2l–n.

The decreasing fiber diameter due to the addition of another material in polymer solution was reported previously^32^ that system, MXene as filler materials were encapsulated by elongated polymer chains that affected the fiber diameter decrease. Furthermore, Fan et.al., studied LiBr salt incorporated in electrospinning solution^40^, which results in an increase in the conductivity of the spinning solution and surface charge density. When the Taylor cone was generated by electric field force, the whipping jets at transition distance were unstable and split into more filaments, making ultrafine fibers. As for the different morphology of ZnT-15, the average fiber diameter increased and presented alignment into the membrane. The larger size distribution was determined from PU/PAN/ZnT-15. This phenomenon is probably due to the high dielectric constant of PU/PANZnT-15 as can be seen in Figure S3. The effect of dielectric constant on alignment fiber was proposed by Sun et al.^41^. The dielectric constant influences the instability function of the available free charge in the solution. Then, the oriented fibers are formed. The dielectric constants of zinc salt-modified fibers are reported in Table S2 and Fig. S3, which reveal that ZnT-15 has the highest dielectric constant, which may be due to the increase in Zn(OTf)_2_ that also increases the charge carriers in the polymer membrane^42^. In addition, the positive correlation between dielectric constant and ionic conductivity was studied and reported by Muchakayala et.al^43^. Interestingly, PU/PAN/ZnT-15 has a fluffy fiber structure, similar to the fluffy 3D structure reported by Juhasz et al. describing the interaction between solvent (DMF) and salt and charge^44^. The preparation process for the membrane is displayed in Fig. 1, which explains the ion transport within the polymer structure through O in urethane (-COONH-) and N in the acrylonitrile (-CN) group^34,45^.

### FT-IR analysis of the Zn salt incoporated PU/PAN electrospun fiber mat chemical structures

**Figure 3:**
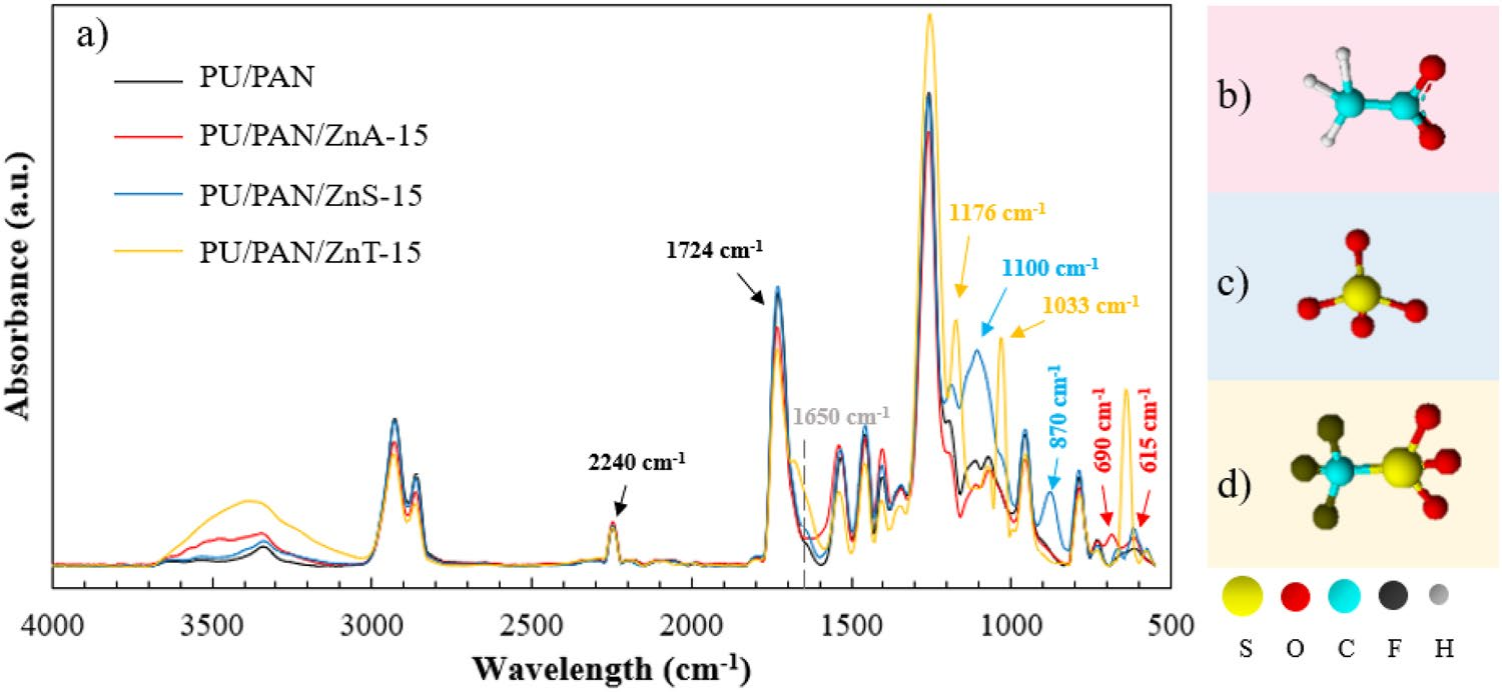
(**a**) FT-IR spectra of PU/PAN and Zn salt incorporated membranes. Chemical structures of the (**b**) SO_4_^2−^, (**c**) CH_3_COO^−^, and (**d**) CF_3_SO_3_^−^ anions.

The chemical structures of Zn salt-incoporated PU/PAN electrospun fiber mats were confirmed by FT-IR, as shown in Fig. 3a. The spectra of the PU/PAN fiber presented broad N–H streching and bending vibrations of the urethane linkage amide group around 3330 and 1528 cm^−1^, respectively. The absorption peak at 2240 cm^−1^ is assigned to the nitrile (−CN) group of the PAN matrix. The occurrence of the characteristic band at 1724 cm^−1^ confirms the C = O stretching bands of the carbonate group in polycarbonate. Meanwhile, the overlap peak at 1724–1670 cm^−1^ can be attributed to the urethane and carbonate carbonyl groups of the PU^28^. The Zn(CH_3_CO_2_)_2_ modified PU/PAN fiber (Fig. 3b) presented peaks at 1067, 690, and 615 cm^−1^, which correspond to the deformation vibration of Zn–O, acetate anion twisting, and scissoring, respectively^21,46^. The results revealed non-apparent bands of chemical bonding from specific sides of the PU/PAN fiber and zinc acetate salt. Next, the ZnSO_4_-modified PU/PAN fibers (Fig. 3c) displayed S = O stretching at 1100 and 870 cm^−1^ from the sulfate ion. The considerable increase of the peak at approximately 1100 cm^−1^ can be observed, probably due to the feature of the modifier being very intensive absorption caused by S = O stretching^47^. Then, the spectrum of the Zn(OTf)_2_ modified PU/PAN fiber (Fig. 3d) showed peaks at 1242 and 1176 cm^−1^ assigned to the symmetric and asymmetric CF^3−^ stretching vibrational modes. The shoulder peak at approximately 1650 cm^−1^ confirms the occurrence of Zn^2+^-bonded C = O^48^. The triflate ions are confirmed by the peak at 1033 cm^−1^ related to the symmetrical stretching vibration mode of SO_3_ [υ_s_ (SO_3_)] and a peak at approximately 640 cm^−1^ corresponding to the bending of C–F and SO_3_ groups and CS symmetrical stretching^21,49^. In addition, the elemental analysis of Zn salt-modified membranes PU/PAN/ZnA-15, PU/PAN/ZnS-15, and PU/PANZnT-15 was performed by elemental mapping and energy dispersive X-ray spectroscopy. In Fig. S5 (a–c), the Zn element is dispersed all over the mapping area, and the C, N, and O elements, the major contents in the polymer chain, can be observed in Fig. S4d–f. In addition, the S element is detected on the membranes ZnS and ZnT, and the F element can be seen in the Zn(OTf)_2_ modified membrane (Fig. S4f). The polar functional group interaction with the salt from the salt-incorporated fiber membranes can support ionic conduction in high-porous polymer electrolytes. Then, some of the functional groups affect other membrane properties to improve electrochemical performance and battery safety.

### Thermal properties of the Zn salt included PU/PAN electrospun fiber mats

**Figure 4:**
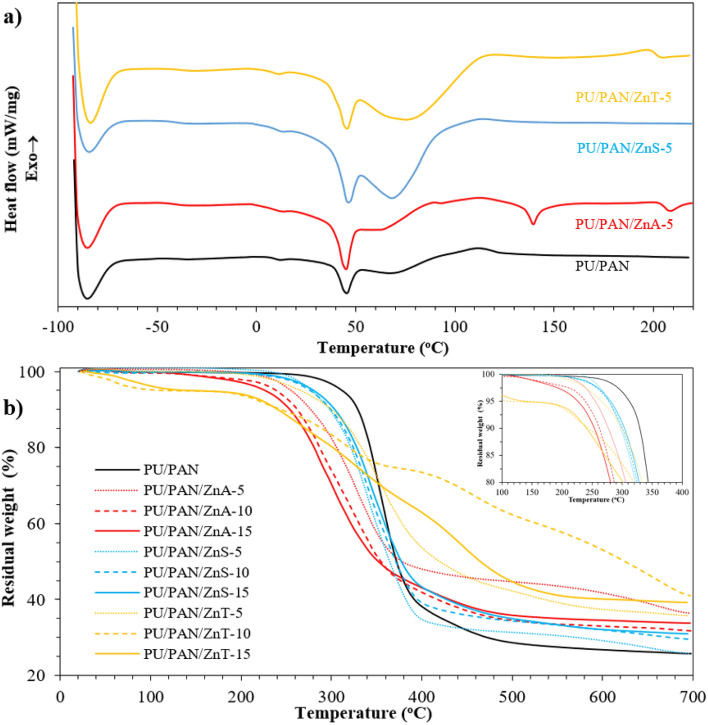
Plot of (**a**) DSC thermograms of (black line) PU/PAN, (red line) PU/PAN/ZnA-5, (blue line) PU/PAN/ZnS-5, and (yellow line) PU/PAN/ZnT-5. Plots of (**b**) TGA for the Zn salt incorporated PU/PAN high porous polymer electrolytes.

Differential scanning calorimetry (DSC) was used to further investigate the microphase separation of the Zn salt using PU/PAN electrospun fiber mats. The thermal behavior of the materials was examined by scanning the samples from –90 °C to 200 °C at a heating rate of 20 °C·min^−1^ under nitrogen on an aluminum pan. The DSC thermograms of the zinc salt-modified PU/PAN electrospun fiber mats with different zinc salts are illustrated in Fig. 4a. The glass transition temperature (T_g_) of PAN fiber was reported to be 100 °C^50^. The PU/PAN sample is presented as line a, which indicates its glass transition temperature (T_g_) to be 7.1 °C and 45.3 °C. The T_g_ of PAN in the PU/PAN sample seems to overlap with the closely endothermic peak at approximately 95.0 °C. This overlapping thermal characteristic of PAN may be because the PAN phase is trapped in the PU phase, and this trapping effect probably enhances the thermal properties in the PU segment. The DSC thermogram of PU/PAN/ZnA-5 presents T_g_ at 3.1 °C and 45.0 °C. The T_g_ of the PAN phase is approximately 90 °C. PU/PAN/ZnA-5 also shows an additional endothermic peak at 139.9 °C. Next, the thermogram of PU/PAN/ZnS-5 presents T_g_ at 8.1 °C and 46.6 °C. The T_g_ of the PAN phase is shown at approximately 95 °C. The thermogram of PU/PAN/ZnT-5 shows T_g_ at 6.1 °C and 45.3 °C. The T_g_ at temperatures around 40–50 °C is similar to the T_g_ hard segment of the polycarbonate-based polyurethane sample in Bao et.al. study^28^. The transition at approximately T_g_ of PAN for PU/PAN/ZnT-5 presents high area merging, which refers to good dissolution of Zn(OTf)_2_ salt into the polymer matrix and the appearance of C–F, which probably increases the amount of H bond in the polymer chain.

In addition, the thermograms of all Zn-salt modified samples present a higher area of endothermic peak at close to 100 °C than PU/PAN, particularly for PU/PAN/ZnS and PU/PAN/ZnT. There is the possibility that the hygroscopic nature of these samples, as expressed by the effect of moisture absorption, leads to good water uptake.

The thermal stability of the fabricated PU/PAN and PU/PAN/ZnX-y salt-incorporated electrospun membranes was evaluated by thermogravimetric analysis at 30 °C–700 °C under N_2_ at a heating rate of 20 °C min^−1^ (Fig. 4b). The plot of the PU/PAN membrane reveals degradation temperature at 5 wt% loss (T_d,5_) at 297 °C, and the weight loss corresponds to high decomposition of the PU/PAN membrane at approximately 350 °C. Then, the decomposition rate is decreased until a char residual of 40 wt% remains. The T_d,5_ values of the Zn(CH_3_CO_2_)_2_ incorporated samples (ZnA-y) seem to decrease to 256 °C, 238 °C, and 226 °C for PU/PAN/ZnA-5, PU/PAN/ZnA-10, and PU/PAN/ZnA-15, respectively. The early decomposition loss is due to the volatile component and water. Meanwhile, the char residual weights of PU/PAN/ZnA-y are higher than those of PU/PAN. Next, the T_d,5_ values of PU/PAN/ZnS-5, PU/PAN/ZnS-10, and PU/PAN/ZnS-15 are 278 °C, 275 °C, and 260 °C, respectively. This decreasing trend of T_d,5_ with increasing Zn salt may be related to the decrease in the polymer:salt ratio, that is, polymer contents. The TGA thermogram of PU/PAN/ZnT presents quite high weight loss at approximately 100 °C, which may be because the absorbed moisture due to its hygroscopic nature evaporated. Nevertheless, the decomposition slope of the PU/PAN/ZnT sample after moisture evaporation seems slower than that of the other samples. There is the possibility that the C–F from Zn(OTf)_2_ delays the degradation process due to the high bonding energy (C–F = 485 J mol^−1^, C−C=347 J mol^−1^) and fluorinated atom. Moreover, the char residual of PU/PAN/ZnT is developed from neat PU/PAN, particularly from the Zn(OTf)_2_ salt addition. Changing degradation temperature and loss of absorbed moisture in the modified fibers have been reported in the literature^51,52^. In the same way, different degradation temperatures between the pristine fiber and the addition of electroactive salt fillers were proposed by Tiwari and Maiti^51^.

**Figure 5:**
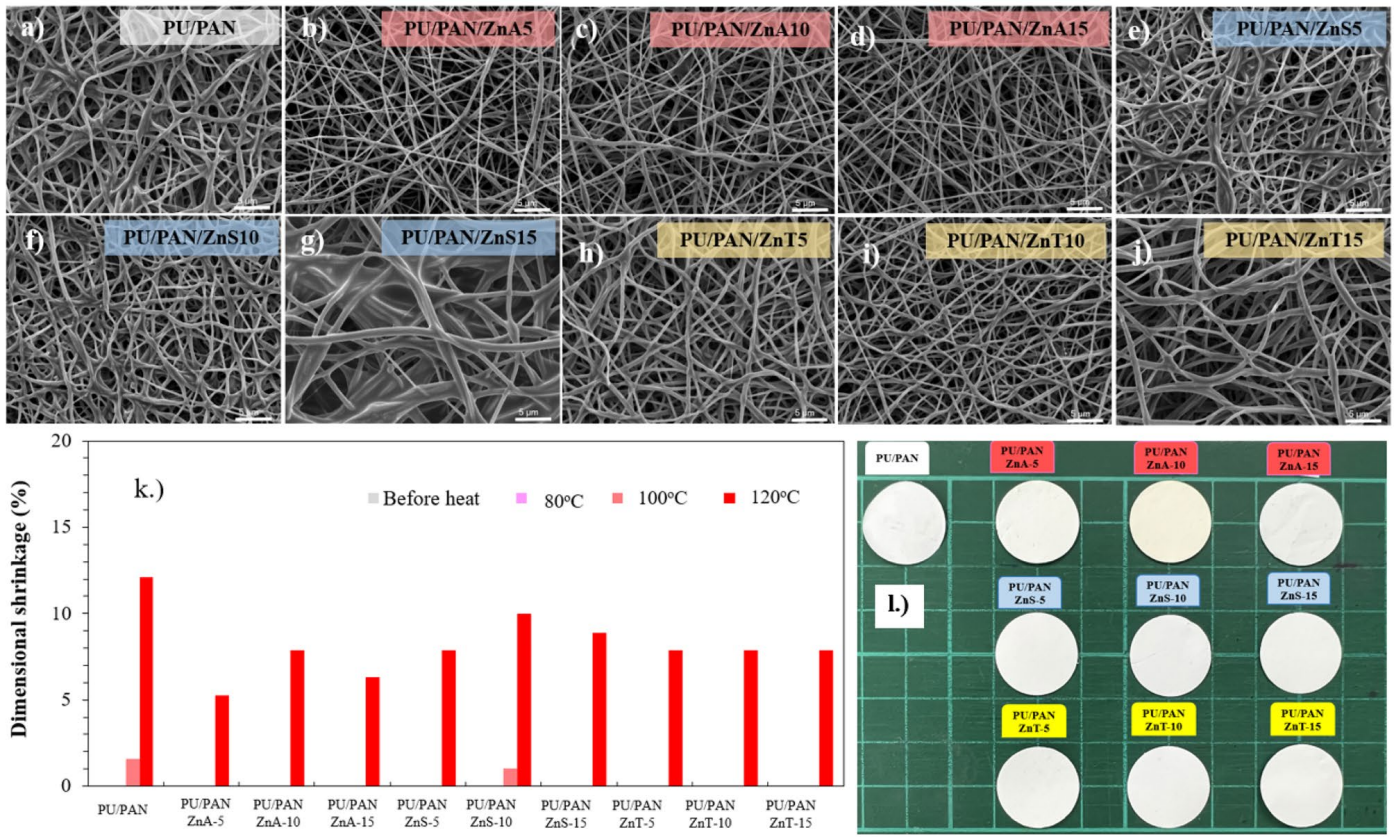
SEM micrographs of (**a**) PU/PAN, (**b**) ZnA-5, (**c**) ZnA-10, (**d**) ZnA-15, (**e**) ZnS-5, (**f**) ZnS-10, (**g**) ZnS-15, (**h**) ZnT-5, (**i**) ZnT-10, and (**j**) ZnT-15 after heat treatment at 120 °C. (**k**) Plot of dimensional shrinkage of high porous polymer electrolytes after heat treatment. (**l**) Representative dimensional stability investigation appearances of high porous polymer electrolytes after heat treatment at 100 °C.

In addition to thermal decomposition, thermal dimensional stability is a crucial thermal property. The shape shrinkage or dimensional change of samples can affect batteries in a bad ways such as electrical short circuits or thermal runaway. Figures S4a and S4b show the highly porous polymer electrolytes heat-treated at 80 °C and 120 °C, respectively. The electrolyte membrane samples were cut into 19 mm diameter circles and brought to heat in an oven. The samples after heat treatment were drawn into 19 mm diameter circle shapes, which equal the original cutting shape for electrolyte membrane applications and measured by a standard scale ruler for dimensional change studies. The morphology after heat treatment is presented in Fig. 5a–j. After 80 °C for 1 h of heat treatment, all samples’ appearances were observed to retain their initial shapes, that is, without shrinkage. Next, continuous heat treatment at 100 °C for another 1 h was performed on the same samples. With higher temperatures and longer times, most polymer electrolyte membranes showed shape resistance to dimensional change; however, the pristine PU/PAN and ZnS-10 were observed to gradually change shape. Finally, after 120 °C of heat treatment, the dimensional change of the unmodified PU/PAN membrane decreased by 12.1%. Percentages of dimensional shrinkage were plotted in Fig. 5k. The dimensional change at 120 °C of the PU/PAN sample is clearly higher than that of other Zn salt-modified samples. The Zn(CH_3_CO_2_)_2_ modified PU/PAN membranes present lower shrinkage than the other membranes; moreover, the SEM images confirm the retained straight fiber shape as shown in Fig. 5b–d. At the same level of heat treatment, the dimensional shrinkage of all ZnT samples was measured to be 7.8%, which is lower than that of PU/PAN. Finally, the ZnSO_4_-modified PU/PAN membranes are observed to have shrinkage of approximately 7.8%–10.0%; however, these values are lower than 12.1% of PU/PAN. Based on the shrinkage results, it is undeniable that the Zn salts clearly enhance the dimensional shrinkage resistance of the membranes. SEM can confirm the different morphologies of the fibers after heat treatment, such as curl and connecting fibers. In addition, the enhancement of shrinkage resistance of the fiber membrane may relate to adding functional salts such as Zn(OTf)_2_. For example, adding the Zn(OTf)_2_ salt acts as a temperature retarder that corresponds to C–F, as we clearly found evidence from the FT-IR results. The sample’s appearance after 100 °C of heat treatment, which is above the high operation temperature of batteries, is shown in Fig. 5l.

### Mechanical properties of Zn salt incorporated PU/PAN electrospun fiber mats

**Figure 6:**
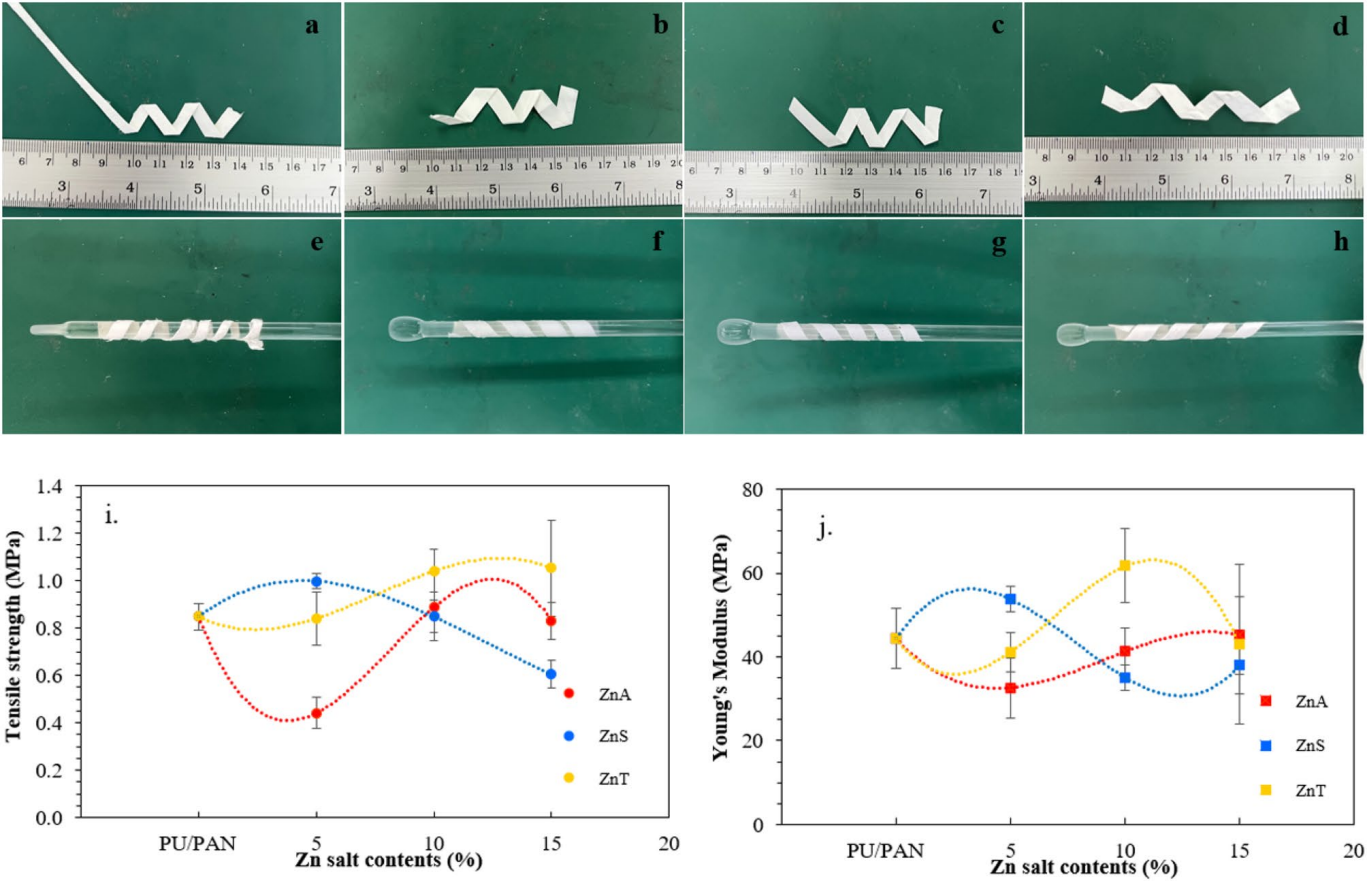
Images of the folding appearances of (**a**) PU/PAN, (**b**) PU/PAN/ZnA-15, (**c**) PU/PAN/ZnS-15, (**d**) PU/PANZnT-15 and (**e**) PU/PAN, (**f**) PU/PAN/ZnA-15, (**g**) PU/PAN/ZnS-15, and (**h**) PU/PAN/ZnT-15. Plots of mechanical properties of the high porous polymer electrolytes: (**i**) tensile strength and (**j**) Young’s modulus.

The mechanical properties of highly porous polymer electrolytes influence the safety of use and large-scale manufacture of batteries. The obvious folding and rolling appearances of highly porous polymer electrolyte membranes are presented in Fig. 6a–h. The high salt ratio sample is folded in several angles with no broken point; moreover, it can be brought to rolling up approximately 1 cm in diameter of the glass rod. These apparent flexibilities indicate their acceptable utility in real-life applications.

In addition, their mechanical properties were evaluated using a universal testing machine. One mechanical characteristic of electrospun membranes is their tensile strength, which determines the membrane's ability to withstand mechanical stress under various applications. In this work, the effects of the addition of Zn salt on the tensile strength and Young’s modulus of the electrospun membranes are exhibited in Fig. 6i,j. As can be observed in these plots, the addition of Zn(CH_3_CO_2_)_2_ in PU/PAN has no clear improvement in the mechanical properties of the membranes. The ZnSO_4_-incorporated PU/PAN membrane presents slightly increasing mechanical properties at 5 wt% ZnSO_4_ in PU/PAN, and then the properties decrease when adding more ZnSO_4_. Both the tensile strength and Young’s modulus of the polymer membrane gradually improve by adding Zn(OTf)_2_ salt. The improvement in the PU/PAN/ZnT samples is probably due to the addition of the functional group, as shown in the FT-IR results, which increases hydrogen bonding in the whole polymer chain.

### Physical properties and water contact angle of high porous polymer electrolytes

**Figure 7:**
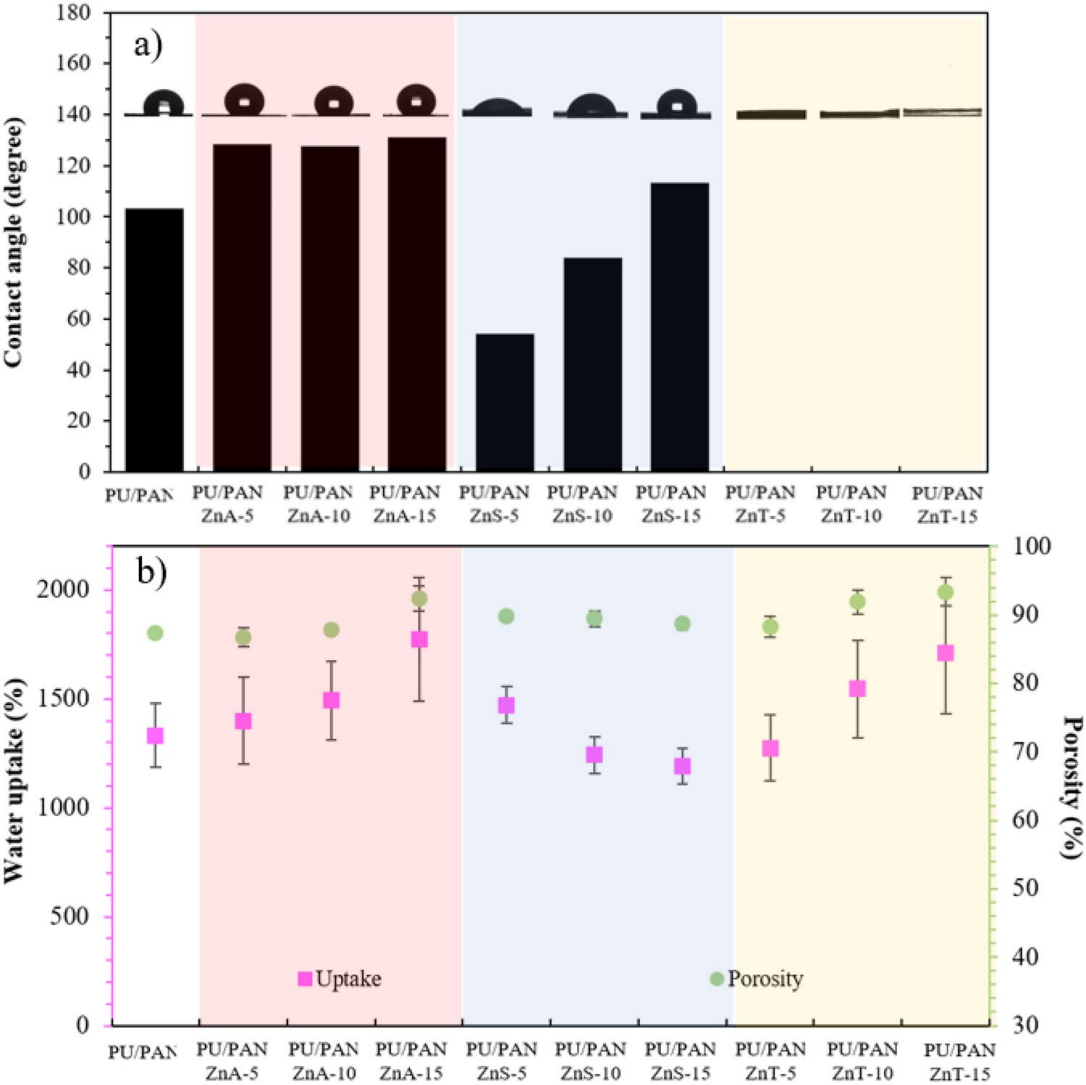
Representative (**a**) contact angle values and (**b**) water uptake (pink square) and porosity (green circle) values of the PU/PAN/Zn salt electrospun membranes.

It is undeniable that the interaction between polymer electrolytes and liquid water is one of the crucial properties for determining the desirable performance of ion movement in polymer electrolytes. Contact angle measurement is a direct way to explain the wettability of our samples. A drop of 2 μL of water was placed on the samples, and within 5 s, the contact angles were recorded. Figure 7a illustrates the contact angles after averaging three measurements at ambient conditions. The angle between the water droplet and the PU/PAN membrane is approximately 103°. All the PU/PAN/ZnA modified samples present a high contact angle compared with PU/PAN without Zn salt, whereas the contact angle is reduced when PU/PAN is incorporated with ZnSO_4_. The contact angle of PU/PAN/ZnS-5 is reduced to 54°. Meanwhile, there is an upward trend in contact angles for PU/PAN/ZnS-10 and PU/PAN/ZnS-15. The increasing trend with increasing amounts of ZnSO_4_ in the ZnS samples is probably due to the agglomeration of the hydrophilic group. In addition, the Zn salts used in this work contain in their structures the hydrophobic −CF_3_ group and the hydrophilic group –SO_3_^−^. In addition, the FT-IR results reveal the increment of the OH stretching group, increasing the hydrophilic group in the PU/PAN/ZnT sample. The more hydrophilic the material is, the higher the interaction with water.

Water uptake, the water absorption ability of a material, is shown in Fig. 7b (pink square sign). The porous polymer electrolyte membrane samples were immersed in deionized water for 24 h, and then the differences in weight before and after water immersion were measured for all samples. The porosity values of the highly porous polymer electrolyte membranes were calculated and are presented in Fig. 7b (green circle sign). The water uptake of PU/PAN is reported to be approximately 1,332%, which is significantly higher than the 100% water uptake of the poly(2,6-dimethyl-1,4-phenylene oxide)-trimethylamine separator for a Zn air system^53^. The PU/PAN/ZnA samples present an increasing amount of liquid uptake with increasing Zn(CH_3_CO_2_)_2_ content, and water uptake is up to 1,773% at the highest Zn(CH_3_CO_2_)_2_ content. In Fig. 7b, this increase in uptake value seems to go hand in hand with increasing porosity in the fiber structure, which is probably related to the decrease in fiber diameter when adding Zn(CH_3_CO_2_)_2_ salt, as shown in Fig. 2. This is because the space area between fibers is enlarged when minimizing fiber diameter size. The membrane including ZnSO_4_ presents a gradual increase in uptake percentages early, and then the uptake decreases when adding more ZnSO_4_.

Like the PU/PAN/ZnA samples, the PU/PAN/ZnT samples show an increasing uptake value when the salt content is increased. The increase in uptake value is related to the increase in porosity of the membrane. To the best of our knowledge, the porosity of a membrane directly affects the reservoir area for liquid uptake because the liquid would fill up between pore structures. Moreover, the high hydrophilicity groups, such as –OH, as shown in Fig. 3, on the fiber surface probably reduce the adsorption time, as explained by the contact angle measurements.

### Ionic conductivities of the high porous polymer electrolytes

**Figure 8:**
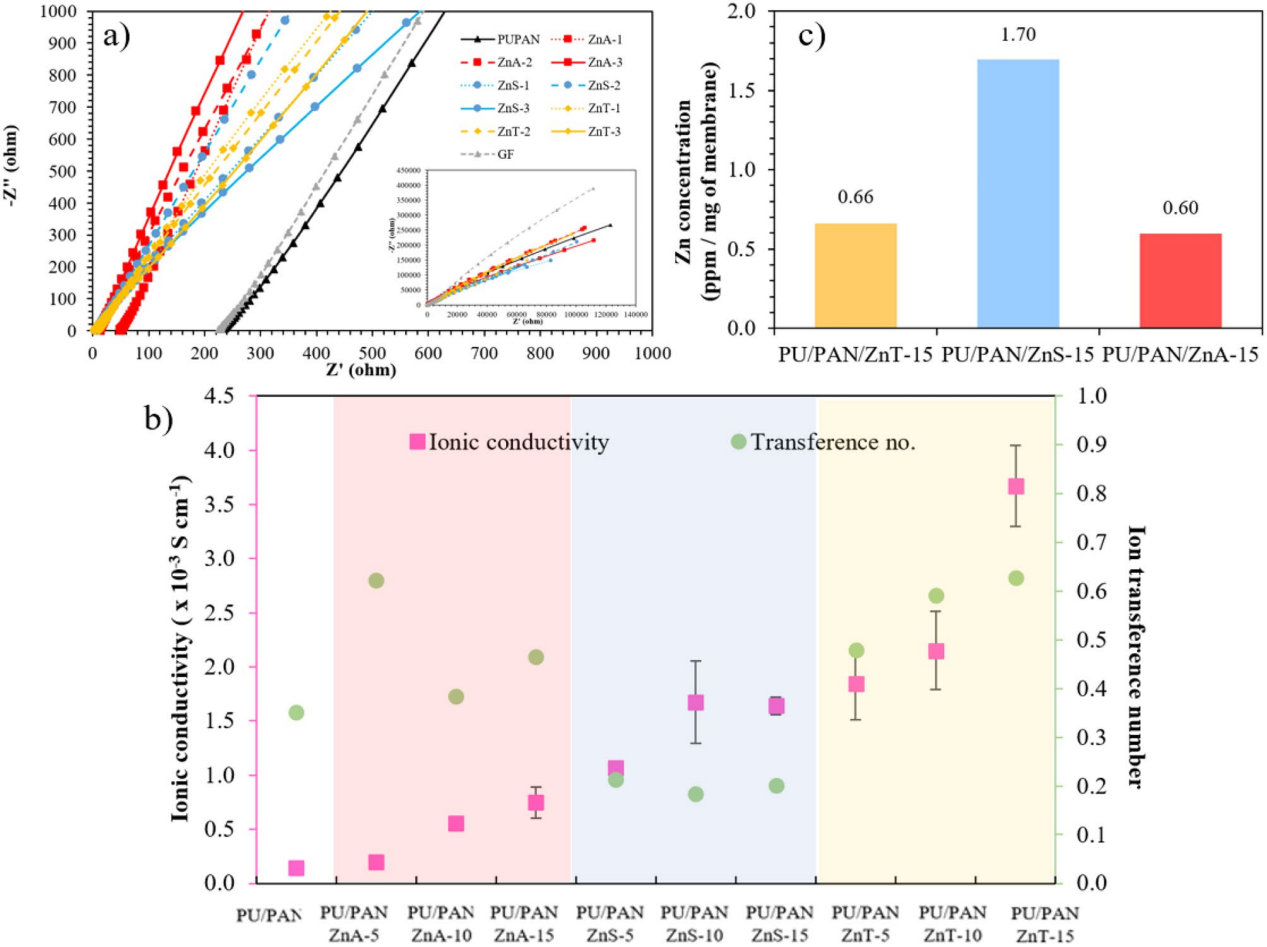
Representative (**a**) Nyquist plots of stainless-steel blocking symmetric cell with high porous membrane electrolyte tested. (**b**) Plot of ionic conductivity and transference number versus sample with different zinc salt incorporated membrane. (**c**) Concentration of dissolution zinc with different zinc salt incorporated membrane.

The real utilization of polymer electrolyte is as a medium between two electrodes. In this position, the highly porous polymer electrolyte should present enough ionic conductivity. The crucial role of high porous polymer electrolytes in energy storage devices is to conduct ion charge migration. EIS usually provides data for real resistance and imaginary capacitance through the ability of a circuit to resist the flow of electrical current. The ionic conductivities of the highly porous polymer electrolyte membranes sandwiched between two stainless steel blocking electrodes were measured by AC impedance spectroscopy under setting conditions of 10 mV from 100 kHz to 0.01. The Nyquist plot, shown in Fig. 8a, presents the impedance relationship between the imaginary part (Z″) on the y axis and the real part (Z′) on the x axis. In the plot, the internal resistance of the testing cell can be clearly observed on the x axis. The unmodified PU/PAN membrane has a higher internal resistance than the other Zn salt membranes. From this plot, it is undeniable that one of the benefits of Zn salts is to reduce membrane resistance. Moreover, the effect of the anion and adding salt is shown on the left-hand side, shifting on the x axis in this plot. The ionic conductivity (δ) of materials can be calculated using Eq. (2). ^54^Testing cells with the high porous polymer electrolyte membrane were tested by EIS, which is presented as the Nyquist plots in Fig. 8a and the ionic conductivity values are presented in Fig. 8b. The intercept at the x axis point represents electrolyte resistance (Rs), which can be used to calculate ionic conductivity (δ) with Eq. (3). The PU/PAN membrane presents the highest ohmic resistances compared with the other membranes and offers the lowest ionic conductivity of 0.140 ± 0.019 mS cm^−1^. This value is close to the conductivity of a conventional system glass fiber membrane in an aqueous electrolyte which was 0.144 mS cm^−1^. The resistance value drastically decreases when the membrane is incorporated with Zn salt. In pink square Fig. 8b, the Zn(CH_3_CO_2_)_2_ incorporated membranes present increasing ionic conductivity: PU/PAN/ZnA-5, PU/PAN/ZnA-10, and PU/PAN/ZnA-15 are 0.196 ± 0.034, 0.557 ± 0.061, and 0.747 ± 0.141 mS cm^−1^, respectively. The ionic conductivities of the PU/PAN/ZnS samples are increased relative to PU/PAN without Zn salt, in the range of 1.072 ± 0.029 to 1.676 mS cm^−1^. However, the trend of ionic conductivity seems to level off at PU/PAN/ZnS-15. This stable value is possibly due to the high zinc ion dissolution, which is consistent to the result of zinc dissolution concentration in Fig. 8c. The membranes modified with Zn(OTf)_2_ have the highest value of 3.671 ± 0.373 mS cm^−1^ taken from PU/PAN/ZnT-15. Based on these results, salt concentration can affect the ionic conductivity of a polymer electrolyte, similar to the explanation by Bao et al.^28^. Moreover, the polar functional group on the polymer chain affects coordination with the alkaline ion to support their ion conduction^55^. As can be seen in the ionic conductivity results, the PU/PAN/ZnS samples represent higher ionic conductivity than all the PU/PAN/ZnA membranes. The enhancement is due to the appeareance of the polar group –S–, as found in the FT-IR results, coordinating with the alkaline Zn ion. In addition, the PU/PAN/ZnT samples, which contain both polar groups of CF and SO_3_, provide higher ionic conductivity than the PU/PAN/ZnS membrane. The ionic conductivity values of this work and some GPEs are compared and shown in Table 1.

**Table 1:** Review of other polymer electrolyte for Zn ion batteries.

System	Ionic conductivity (S cm^-1^)	Temperature (^o^C)	References
Precursor monomer to build in-situ polymerized SPEs Poly (ethylene oxide) (PEO)-based SPE	2.87 $\times$ 10^–5^ 8.33 $\times$ 10^–4^	30 °C 60 °C	^16^
Polyethylene glycol (PEG)-based electrolytes composed with zinc acetate Zn(CH_3_COO)_2_	1.55 $\times$ 10^–6^	30 °C	^23^
Carboxymethyl cellulose (CMC) GPE with Zn(CH_3_COO)_2_ salt	2.10 $\times$ 10^–3^	RT	^21^
EMITf/PVDF-HFP polymer electrolyte (1-ethyl-3-methylimidazolium Trifluoromethanesulfonated (EMITf) incorporated poly (vinylidene fluoride-hexafluoropropylene) (PVdF-HFP))	1.44 $\times$ 10^–4^	RT	^56^
PU/PAN/ZnT-15 Polyurethane/Polyacrylonitile/Zn(OTf)_**2**_	3.67 $\times$ 10^–3^	RT	This study

Next, the ion transference number (t_Zn_^2+^), green circle in Fig. 8b, was characterized by assembling the Zn symmetrical cell and calculating the result by using Eq. (3). The ion transference number is the contribution of Zn^2+^ as an cation to the overall ionic conductivity, which implies concentration polarization^38^. The DC polarization current versus time plot is presented in Fig. S6. From the calculated transference numbers, the membranes incorporated with Zn(CH_3_CO_2_)_2_ and Zn(OTf)_2_ present higher transference numbers than the pristine PU/PAN, indicating that the ionic conductivities of PU/PAN/ZnA, and PU/PAN/ZnT have contributed more Zn^2+^ ions than pristine PU/PAN. Meanwhile, the ZnSO_4_ modified membrane has the lowest ion transference number. The possible reasons for the enhancement of the ion transference number of Zn(CH_3_CO_2_)_2_ and Zn(OTf)_2_ may be due to the size of the anion molecule: a bulky anion can decrease the coordination number of H_2_O surrounding the Zn^2+^ ion, resulting in improved ionic conductivity^11^. Figure 8c presents the result of zinc dissolution concentration, which can be observed by the high amount of zinc concentration in the PU/PAN/ZnS samples. This result is consistent with the high interaction between water molecules and ZnSO_4_. Moreover, these bulky anions are supposed to hinder themselves in transportation, which eventually results in increasing the cation ratio for overall ionic conductivity. Conversely, SO_4_^2−^ (in ZnS) is a compact anion, which is easier to transport and can be counted in overall ionic conduction.

**Figure 9:**
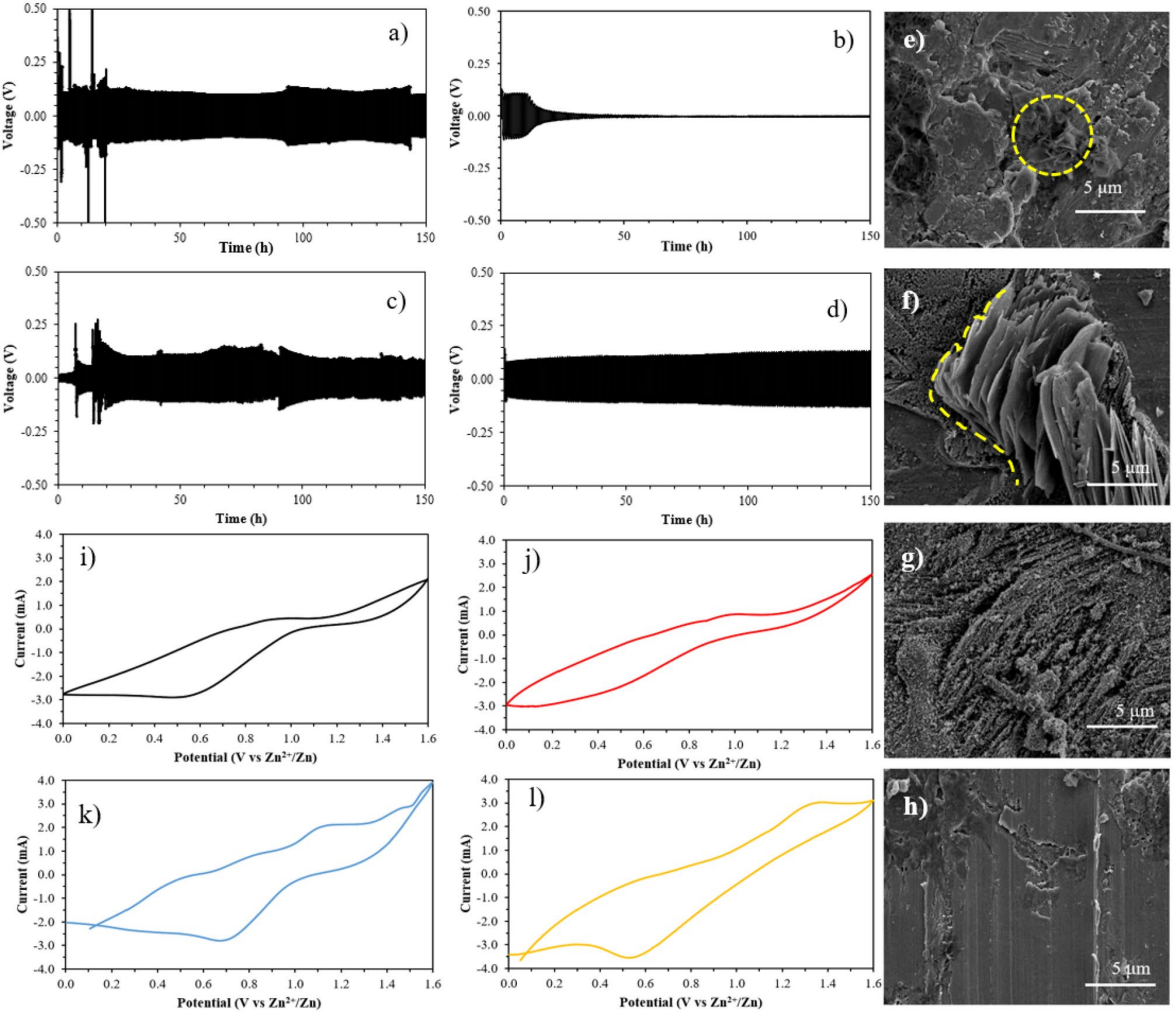
Charge–discharge plots of (**a**) PU/PAN, (**b**) PU/PAN/ZnA-15, (**c**) PU/PAN/ZnS-15, and (**d**) PU/PAN/ZnT-15. zinc foil after testing with (**e**) PU/PAN and (**f**) PU/PAN/ZA-15 (**g**) PU/PAN/ZS-15 (**h**) PU/PAN/ZT-15 polymer electrolyte. CV plots of full cell (NVO//Zn) with the high porous polymer electrolyte membranes: (**i**) PU/PAN, (**j**) PU/PAN/ZnA-15, (**k**) PU/PAN/ZnS-15, and (**l**) PU/PAN/ZnT-15.

The cycling charge–discharge plots at room temperature of pristine PU/PAN and Zn salt-modified PU/PAN are displayed in Figs. 9a–d and S7a–f. The symmetrical (Zn//Zn) cells were long-term tested with a current density of 0.25 mA cm^−2^, and then the responding voltage signals were plotted versus testing time. In Fig. 9a, the voltage profile of pristine PU/PAN shows quite a fluctuation signal in the first 20 h, and then the signal becomes stable; however, the polarized voltage is presented after 100 h. The PU/PAN/ZnA and PU/PAN/ZnS samples show some fluctuation signals during the long-run testing; in addition, the highest unstable signal is observed at over 50 cycles from PU/PAN/ZnA-10. The Zn(CH_3_CO_2_)_2_ in PU/PAN/ZnA has produced an acidic-like electrolyte from saturated water, and the acidic condition affects Zn anode dissolution and corrosion, which may have a negative effect on battery performance^57^. Of these samples, only deionized water uptake may not be enough for smooth electrochemical stability. Finally, the voltage profile of the PU/PAN/ZnT samples is observed in Figs. S6e–f, displaying a less fluctuating signal than any other samples. These constant voltage profiles reveal the exceptional Zn plating/stripping and unchanging within the internal of the operating cell, which may result from high ionic conductivity and ion transference number of the PU/PAN/ZnT sample^12^. Moreover, the SEM image verification of zinc electrode in Fig. 9e–h confirms that the zinc electrode assembling with PU/PAN and PU/PAN/ZnA-15 contains some zinc dendrite structure, whereas the zinc electrode assembling with PU/PAN/ZnT-15 present a smoother surface. The outstanding result in Zn plating/stripping of Zn(CF_3_SO_3_)_2_ has been reported in liquid salt electrolyte^7,57^.

To understand the redox reaction, CV can be performed in three electrode formations to understand the redox reaction of electrochemical cells, where the potential is supplied between a reference and working electrode and the current is measured between the working and counter electrodes. The effects of highly porous polymer electrolyte membranes were evaluated from the cyclic voltammograms using the full-cell vanadium based (NVO) cathode and Zn anode (NVO//Zn). The SEM elemental mapping of NVO coated on graphite paper was utilized to confirm the cathode material as Fig. S8a–e. The experiments were performed at a 10 mV s^−1^ scan rate. The highest Zn salt contents (15% w/w) from each Zn salt modified membrane were tested and reported in Fig. 9i–l. Figure 9g,h, PU/PAN and PU/PAN/ZnA-15 show an unclear peak of the oxidation reaction, but it seems to reveal a small peak at around 1.0 V. While PU/PAN/ZnS-15 and PU/PAN/ZnT-15 (Fig. 9k,l) clearly present an oxidation peak at around 1.1 and 1.3 V, respectively, which is related to the extraction of zinc ion. However, the peaks that represent the reduction reaction are around 0.5–0.7 V for all samples, indicating the insertion of zinc ions on the electrode. The complete CV cycle indicates the plenary cell reaction. However, the insufficient charge carrier of the pristine PU/PAN membrane shows an unclear oxidation reaction, as shown in the CV curve. Besides, the rate performance of the samples was tested at different current densities from 0.1 to 5.0 A g^−1^ for full cell battery Zn/Polymer electrolyte/NVO. In Fig. S9, the average capacity at 0.1 A g^−1^ was 335 and 120 mAh g^-1^ for cell assembled with PU/PAN/ZnT15 and PU/PAN, respectively. The sufficient ion carrier part of PU/PAN/ZnT15 may be the cause of its higher capacity than PU/PAN. However, the plots shows capacity fading after current density returns to 0.2 A g^−1^ due to the morphology change and loss of contact of active material to the carbon substrate^58^.

## Conclusions

The Zn salt incorporated PU/PAN electrospinning fiber membranes were manufactured by electrospinning. The Zn salts used in this work, (Zn(CH_3_CO_2_)_2_), (ZnSO_4_), and (Zn(OTf)_2_), resulted in enhancements in porous morphology, thermal shrinkage properties, and ionic conductivity. The effects of Zn salt can determine the fiber morphology and porous structure, as we proposed in this study, which explains that high dielectric constant salt and solvent provide good fiber producibility. The addition of various Zn salts affected the interaction between fiber and a small amount of water; especially, Zn(OTf)_2_ drastically improved the wettability of the membrane, as presented in contact angle measurements. The crucial parameter of polymer electrolyte, ionic conductivity, was improved with adding Zn salt, and it can be suggested that the addition of Zn salt increases Zn^2+^ as charge carriers. However, the amount of ZnSO_4_ in the PU/PAN membrane shows a decrease in ion transference number due to the effect of compact molecules from sulfate ions. Also, the Zn symmetrical charge–discharge (stripping/plating) could confirm the stable signal from the Zn(OTf)_2_ membrane. Especially the CV plot of the PU/PAN/ZnT-15 membrane provides a good circle of redox reactions. Based on this study, the Zn(OTf)_2_ membrane shows promise as a polymer electrolyte as it provides better performance for use as a water-uptake polymer electrolyte than the pristine membrane and other Zn salts. Promising PU/PAN/ZnT membranes or Zn(OTf)_2_ incorporated in other materials should be developed further in future work.

### Supplementary Information

Supplementary Information.

## Data Availability

The datasets used and/or analysed during the current study available from the corresponding author on reasonable request.

## References

[1] Gür TM (2018). Review of electrical energy storage technologies, materials and systems: challenges and prospects for large-scale grid storage. Energy Environ. Sci..

[2] Hua Y (2020). Sustainable value chain of retired lithium-ion batteries for electric vehicles. J. Power Sources.

[3] Verma D (2022). Blockchain technology and AI-facilitated polymers recycling: Utilization, realities, and sustainability. Polym. Compos..

[4] Obama B (2017). The irreversible momentum of clean energy. Science.

[5] Okhawilai M, Pattananuwat P (2021). Sustainable electroactive materials for energy storage. Curr. Opin. Green Sustain. Chem..

[6] Wang G (2022). Sulfonated polybenzothiazole cathode materials for Na-ion batteries. Chem Commun..

[7] Yu P (2019). Flexible Zn-Ion batteries: Recent progresses and challenges. Small.

[8] Huang J, Cao Y, Cao M, Zhong J (2021). Improving the capacity of zinc-ion batteries through composite defect engineering. RSC Adv..

[9] Nitta N, Wu F, Lee JT, Yushin G (2015). Li-ion battery materials: Present and future. Mater. Today.

[10] Hwang JY, Myung ST, Sun YK (2017). Sodium-ion batteries: Present and future. Chem. Soc. Rev..

[11] Ye Z (2020). Advances in Zn-ion batteries via regulating liquid electrolyte. Energy Stor. Mater..

[12] Zhang T (2020). Fundamentals and perspectives in developing zinc-ion battery electrolytes: A comprehensive review. Energy Environ. Sci..

[13] Zhang N (2020). Materials chemistry for rechargeable zinc-ion batteries. Chem. Soc. Rev..

[14] Yin X (2023). Advanced separator engineering strategies for reversible electrochemical zinc storage. J. Solid State Electrochem..

[15] Zhao C (2021). The strategies of boosting the performance of highly reversible zinc anodes in zinc-ion batteries: recent progress and future perspectives. Sustain. Energy Fuels.

[16] Zhao Z (2021). In-situ formed all-amorphous poly (ethylene oxide)-based electrolytes enabling solid-state Zn electrochemistry. Chem. Eng. J..

[17] Dueramae I, Okhawilai M, Kasemsiri P, Uyama H, Kita R (2020). Properties enhancement of carboxymethyl cellulose with thermo-responsive polymer as solid polymer electrolyte for zinc ion battery. Sci. Rep..

[18] Lu K, Jiang T, Hu H, Wu M (2020). Hydrogel electrolytes for quasi-solid zinc-based batteries. Front. Chem..

[19] Wang G (2023). Sulfonated graphene oxide doped sulfonated polybenzothiazoles for proton exchange membrane fuel cells. J. Membr. Sci..

[20] Wang G (2023). Sulfonated polybenzothiazoles containing hexafluoroisopropyl units for proton exchange membrane fuel cells. Macromolecules.

[21] Dueramae I, Okhawilai M, Kasemsiri P, Uyama H (2021). High electrochemical and mechanical performance of zinc conducting-based gel polymer electrolytes. Sci. Rep..

[22] Cao J (2021). Oxygen defect enriched (NH4)2V10O25·8H2O nanosheets for superior aqueous zinc-ion batteries. Nano Energy.

[23] Polu AR, Kumar R, Joshi GM (2013). Effect of zinc salt on transport, structural, and thermal properties of PEG-based polymer electrolytes for battery application. Ionics.

[24] Hoang Huy VP, So S, Hur J (2021). Inorganic fillers in composite gel polymer electrolytes for high-performance lithium and non-lithium polymer batteries. Nanomaterials.

[25] Li Z, Li X, Jiang Y, Ding Q, Han W (2021). Nanocellulose composite gel with high ionic conductivity and long service life for flexible zinc-air battery. Polym. Test..

[26] Croce F, Appetecchi GB, Persi L, Scrosati B (1998). Nanocomposite polymer electrolytes for lithium batteries. Nature.

[27] Tsutsumi H (2000). Conductivity enhancement of polyacrylonitrile-based electrolytes by addition of cascade nitrile compounds. J. Power Sources.

[28] Bao J (2018). Polycarbonate-based polyurethane as a polymer electrolyte matrix for all-solid-state lithium batteries. J. Power Sources.

[29] Liu Y (2017). Gel Polymer Electrolyte Based on Poly(vinylidene fluoride)/Thermoplastic Polyurethane/Polyacrylonitrile by the Electrospinning Technique. J. Phys. Chem. C.

[30] Zhu M (2019). Multifunctional polymer electrolyte improving stability of electrode-electrolyte interface in lithium metal battery under high voltage. J. Membr. Sci..

[31] Xue J, Wu T, Dai Y, Xia Y (2019). Electrospinning and electrospun nanofibers: Methods, materials, and applications. Chem. Rev..

[32] Tafur JP, Fernández Romero AJ (2014). Electrical and spectroscopic characterization of PVdF-HFP and TFSI—ionic liquids-based gel polymer electrolyte membranes: Influence of ZnTf2 salt. J. Membr. Sci..

[33] Li X (2023). High performance porous poly(ethylene oxide)-based composite solid electrolytes. Chem. Eng. J..

[34] Pham M-N (2022). Acylamino-functionalized crosslinker to synthesize all-solid-state polymer electrolytes for high-stability lithium batteries. Chem. Eng. J..

[35] Chen, P. *et al.* An Artificial Polyacrylonitrile Coating Layer Confining Zinc Dendrite Growth for Highly Reversible Aqueous Zinc-Based Batteries. *Adv Sci (Weinh)* **8**, e2100309 (2021). 10.1002/advs.202100309 10.1002/advs.202100309 PMC8188195 34105273

[36] Likitaporn C (2022). High electrolyte uptake of MXene integrated membrane separators for Zn-ion batteries. Sci. Rep..

[37] Saisangtham S (2022). Polyacrylonitrile/bio-based polyurethane electrospun fiber mats as advanced separators for high-performance Zn-ion batteries. Express Polym. Lett..

[38] Zheng Z (2022). Low-cost zinc-alginate-based hydrogel-polymer electrolytes for dendrite-free zinc-ion batteries with high performances and prolonged lifetimes. Polymers.

[39] Pożyczka K, Marzantowicz M, Dygas JR, Krok F (2017). Ionic Conductivity and Lithium Transference Number of Poly(ethylene oxide):LiTFSI System. Electrochim. Acta.

[40] Fan L, Xu Y, Zhou X, Chen F, Fu Q (2018). Effect of salt concentration in spinning solution on fiber diameter and mechanical property of electrospun styrene-butadiene-styrene tri-block copolymer membrane. Polymer.

[41] Sun Z, Deitzel JM, Knopf J, Chen X, Gillespie JW (2012). The effect of solvent dielectric properties on the collection of oriented electrospun fibers. J. Appl. Polym. Sci..

[42] Liu J, Khanam Z, Muchakayala R, Song S (2020). Fabrication and characterization of Zn-ion-conducting solid polymer electrolyte films based on PVdF-HFP/Zn(Tf)2 complex system. J. Mater. Sci. Mater. Electron..

[43] Muchakayala R, Song S, Gao S, Wang X, Fan Y (2017). Structure and ion transport in an ethylene carbonate-modified biodegradable gel polymer electrolyte. Polym. Test..

[44] Juhasz AG, Molnar K, Idrissi A, Jedlovszky-Hajdu A (2020). Salt induced fluffy structured electrospun fibrous matrix. J. Mol. Liq..

[45] Li X (2023). Poly (ethylene oxide) based solid polymer electrolyte improved by multifunctional additives of poly (acrylamide) and LiI. Electrochim. Acta.

[46] Kavya Valsan E, John A, Raghavendra M, Ravikumar HB (2020). Free Volume Controlled Ionic Conductivity in Poly Vinyl Alcohol/Zinc Acetate Solid Polymer Electrolytes. J. Electrochem. Soc..

[47] Sedlarik V, Galya T, Sedlarikova J, Valasek P, Saha P (2010). The effect of hydrolysis degree on the properties of antibacterial polymeric films based on poly(vinyl alcohol) and zinc sulphate for biomedical applications. J. Biomater. Sci. Polym. Ed.

[48] Sownthari K, Suthanthiraraj SA (2013). Synthesis and characterization of an electrolyte system based on a biodegradable polymer. Express Polym. Lett..

[49] Prasanna CMS, Suthanthiraraj SA (2015). Electrical, structural, and morphological studies of honeycomb-like microporous zinc-ion conducting poly (vinyl chloride)/poly (ethyl methacrylate) blend-based polymer electrolytes. Ionics.

[50] Ding R, Wu H, Thunga M, Bowler N, Kessler MR (2016). Processing and characterization of low-cost electrospun carbon fibers from organosolv lignin/polyacrylonitrile blends. Carbon.

[51] Tiwari S, Maiti P (2023). Salts induced piezoelectric effect in electrospun PVDF based nanohybrids for efficient energy harvesting. Nano-Struct. Nano-Objects..

[52] Zhang H, Zhang T, Qiu Q, Qin X (2021). Quaternary ammonium salt–modified polyacrylonitrile/polycaprolactone electrospun nanofibers with enhanced antibacterial properties. Text. Res. J..

[53] Abbasi A, Hosseini S, Somwangthanaroj A, Mohamad AA, Kheawhom S (2019). Poly(2,6-dimethyl-1,4-phenylene oxide)-based hydroxide exchange separator membranes for zinc-air battery. Int. J. Mol. Sci..

[54] Wu H-Y (2021). Polyoxometalate driven dendrite-free zinc electrodes with synergistic effects of cation and anion cluster regulation. J. Mater. Chem. A.

[55] Zhu X (2021). Strategies to Boost Ionic Conductivity and Interface Compatibility of Inorganic - Organic Solid Composite Electrolytes. Energy Stor. Mater..

[56] Liu J, Ahmed S, Khanam Z, Wang T, Song S (2020). Ionic Liquid-Incorporated Zn-Ion Conducting Polymer Electrolyte Membranes. Polymers (Basel).

[57] Zhang X (2022). Comprehensive review on zinc-ion battery anode: Challenges and strategies. InfoMat.

[58] Senguttuvan P (2016). A High Power Rechargeable Nonaqueous Multivalent Zn/V2O5 Battery. Adv. Energy Mater..

