# Supplementary material for: Zn salts incorporated polyurethane/polyacrylonitrile electrospinning fiber membrane for high porosity polymer electrolyte in Zn ion battery
Source: Sci Rep. 2023 Oct 5;13:16774. doi: 10.1038/s41598-023-43962-6 (PMC10556048; doi:10.1038/s41598-023-43962-6)
Supplement: Supplementary file 1 — Supplementary Information. [file 41598_2023_43962_MOESM1_ESM.doc]

**Zn salts incorporated polyurethane/polyacrylonitrile electrospinning fiber membrane for high porosity polymer electrolyte in Zn ion battery.**

Chutiwat Likitaporn^1^, Manunya Okhawilai ^1,2,3*^, Nangan Senthilkumar ^2^, Tawan Wongsalam^2^, Nattapon Tanalue^4^, Pornnapa Kasemsiri^5^, Jiaqian Qin^2,3^,, Hiroshi Uyama^6^

^1^ Nanoscience and Technology Interdisciplinary Program, Graduate School, Chulalongkorn University, Bangkok 10330, Thailand

^2^ Metallurgy and Materials Science Research Institute, Chulalongkorn University, Bangkok, 10330, Thailand

^3^ Center of Excellence in Responsive Wearable Materials, Chulalongkorn University, Bangkok, 10330, Thailand

^4^ Multidisciplinary Program in Petrochemistry and Polymer Science, Faculty of Science, Chulalongkorn University

^5^ Sustainable Infrastructure Research and Development Center and Department of Chemical Engineering, Faculty of Engineering, Khon Kaen University, Khon Kaen, 40002, Thailand

^6^ Department of Applied Chemistry, Graduate School of Engineering, Osaka University, Osaka 565-0871, Japan.

* Corresponding Author E-mail address: Manunya.o@chula.ac.th

**Preparation of cathode**

**1. Materials**

NH_4_VO_3_ (purity > 99.0%), thiourea (purity > 99.0%) were purchased from Carlo Erba Co. Ltd., poly-(vinylidene fluoride) (PVDF; HSV 900) was received from Kynar. All of the reagents were of analytical purity and used as received.

**2. Method**

In order to prepare the (NH_4_)_2_V_10_O_25_⋅8H_2_O (NVO), the synthesis method was brought from Cao et al. study [1]. Firstly, 0.468 g (4 mmol) of NH_4_VO_3_ powder was dissolved in 50 mL deionized water at 70 °C. Secondly, 0.228g (3 mmol) of thiourea was added into the solution, and it was thoroughly dissolved by stirring. Thirdly, using diluted sulfuric acid, the pH of the solution was adjusted until it reached 2, then it was continued stirred for 30 minutes. Fourthly, the mixture was maintained at 90 °C and stirred for another 2.5 h in an oil bath, a dark green solution was obtained. After the temperature naturally cooled to room temperature, the products were collected and washed with deionized water and ethanol. Then, the final black powder of NVO with rich oxygen defects were obtained after drying at 60°C for 24 h in vacuum. Finally, the NVO cathode was coated on graphite paper by mixing the slurry of NVO, conductive carbon and PVDF with the mass ratio of 7:2:1, respectively.


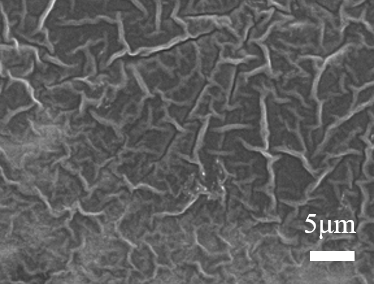

**Figure S1.** SEM image of PU electrospun membrane.


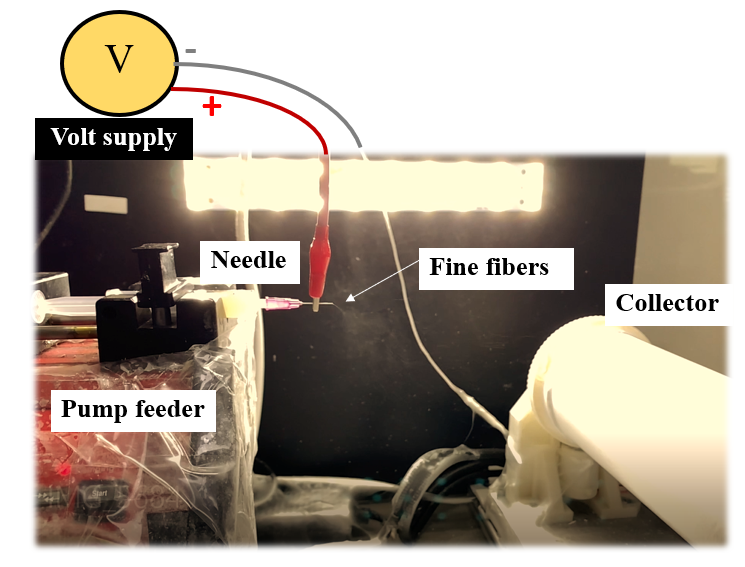


**Figure S2.** Image of electrospinning equipment setting**.**

**Table S1.** Plot of size distribution of entire PU/PAN/ZnX-y samples.

| **No.** | **Matrix fiber** | **Zn salt**  **[Percentage]** | | **Size distribution** | |
| --- | --- | --- | --- | --- | --- |
| 1 | PU/PAN | - | 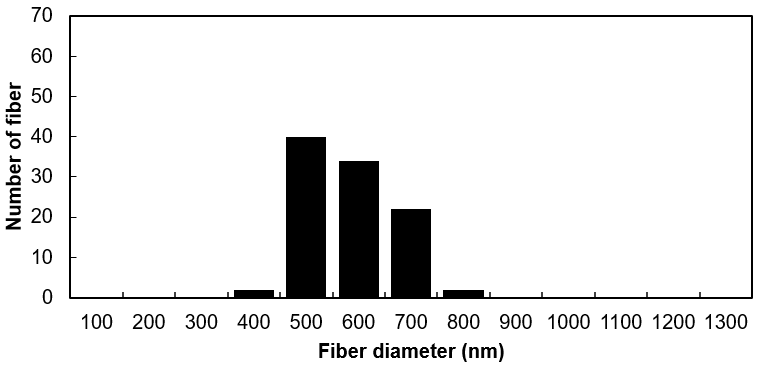 | |  |
| 2 | PU/PAN | Zn(OAc)_2_  [5%wt] | 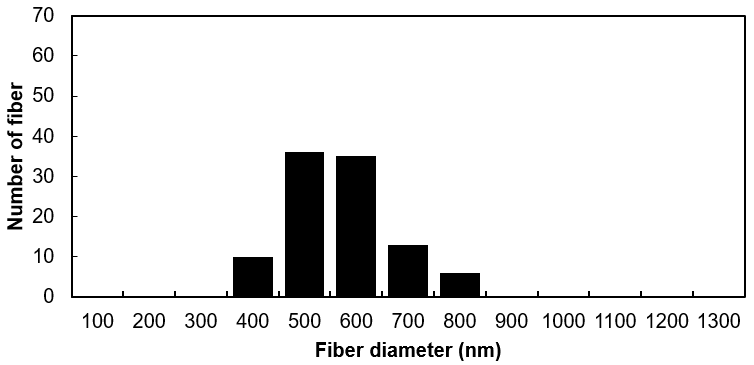 | |  |
| 3 | PU/PAN | Zn(OAc)_2_  [10%wt] | 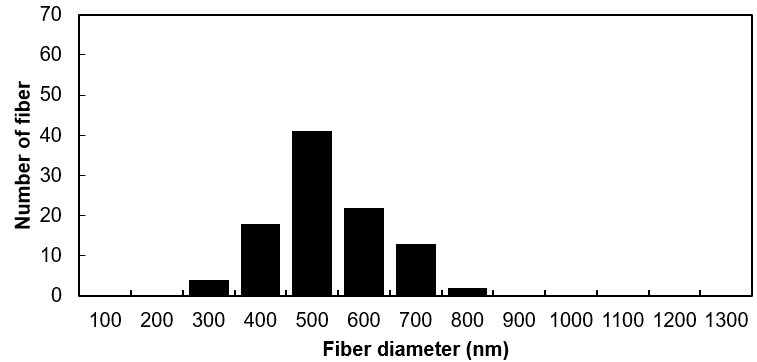 | |  |
| 4 | PU/PAN | Zn(OAc)_2_  [15%wt] | 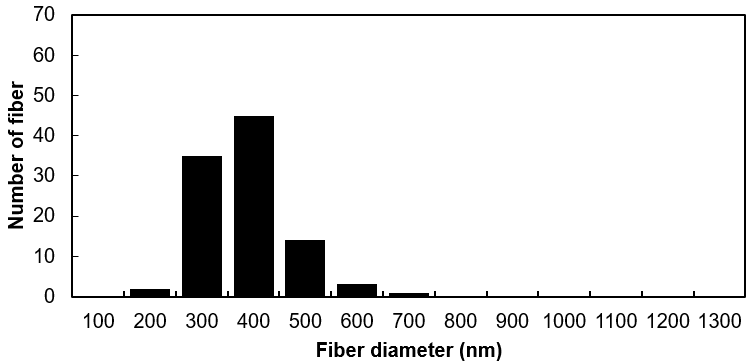 | |  |
| 5 | PU/PAN | ZnSO_4_  [5%wt] | 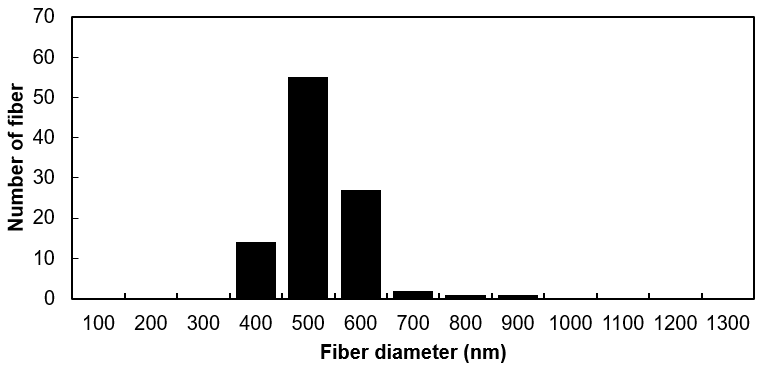 | |  |
| 6 | PU/PAN | ZnSO_4_  [10%wt] | 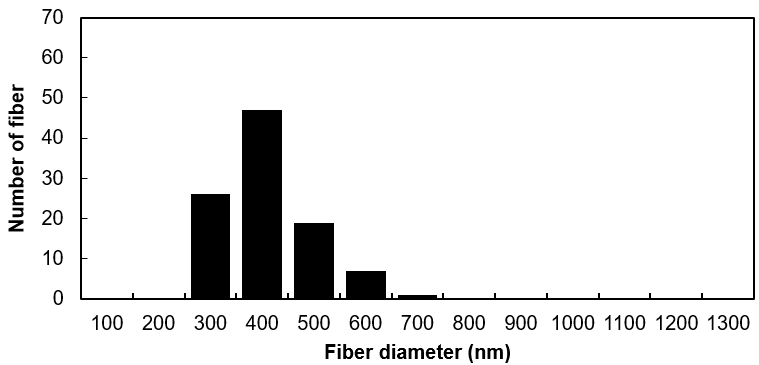 | |  |
| 7 | PU/PAN | ZnSO_4_  [15%wt] | 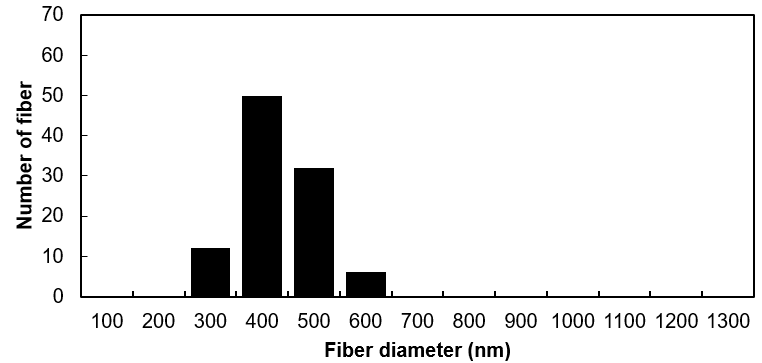 | |  |
| 8 | PU/PAN | Zn(OTf)_2_  [5%wt] | 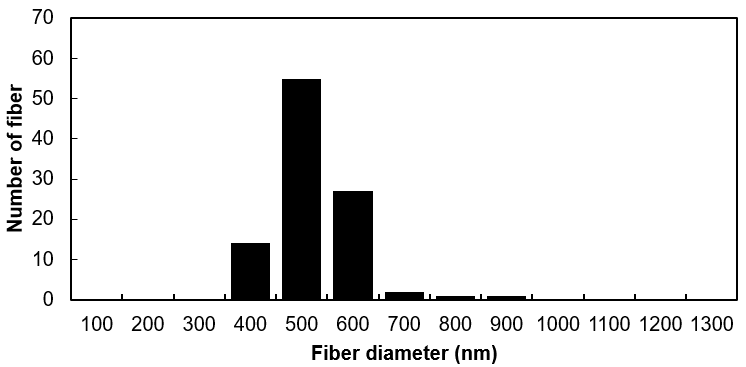 | |  |
| 9 | PU/PAN | Zn(OTf)_2_  [10%wt] | 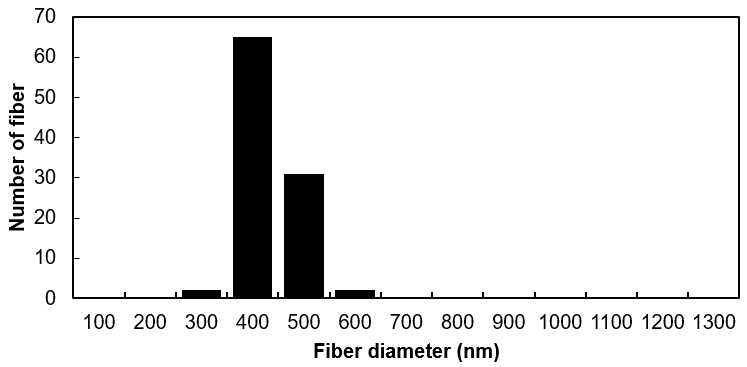 | |  |
| 10 | PU/PAN | Zn(OTf)_2_  [15%wt] | 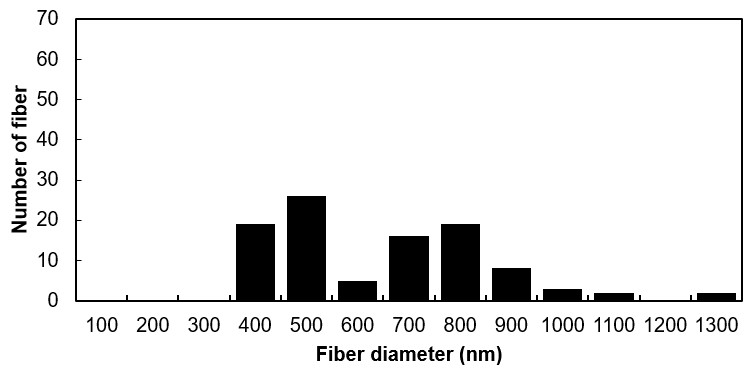 | |  |


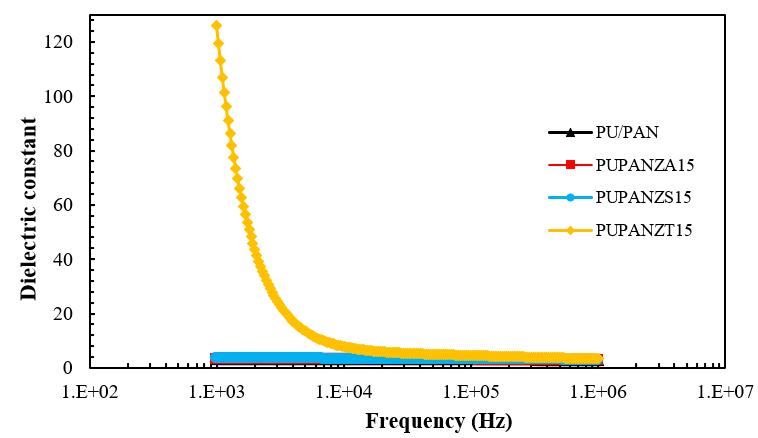


**Figure S3.** Plot of dielectric constant of PU/PAN, PU/PAN/ZnA-15, PU/PAN/ZnS-15, and PU/PAN/ZnT-15.

**Table S2.** Dielectric constant of PU/PAN, PU/PAN/ZnA-15, PU/PAN/ZnS-15, and PU/PAN/ZnT-15.

| **Sample** | **Dielectric constant @ 1 kHz** |
| --- | --- |
| PU/PAN | 3.2 |
| PU/PAN/ZnA-15 | 3.1 |
| PU/PAN/ZnS-15 | 3.4 |
| PU/PAN/ZnT-15 | 126.0 |


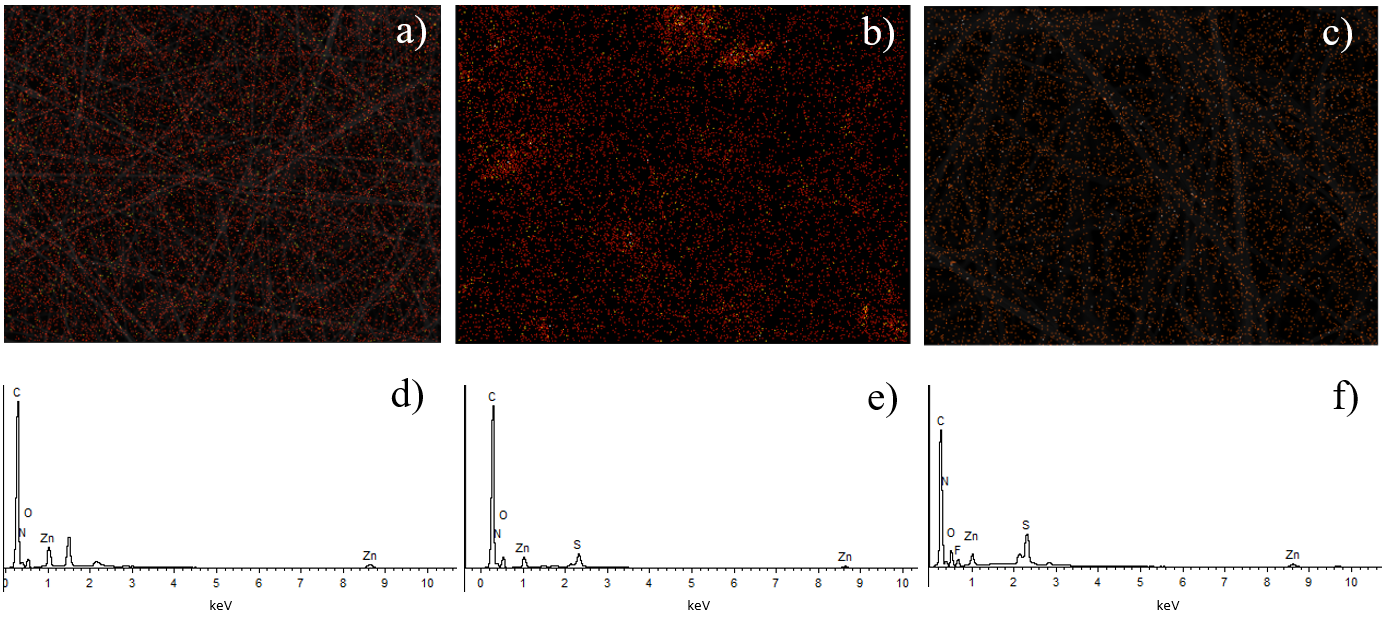


**Figure S4.** Representative of SEM Zn elemental mapping of a) PU/PAN/ZnA-15, b) PU/PAN/ZnS-15, and c) PU/PAN/ZnT-15. Elemental analysis from energy dispersive X-ray spectroscopy (EDS) of d) PU/PAN/ZnA-15, e) PU/PAN/ZnS-15, and f) PU/PAN/ZnT-15.


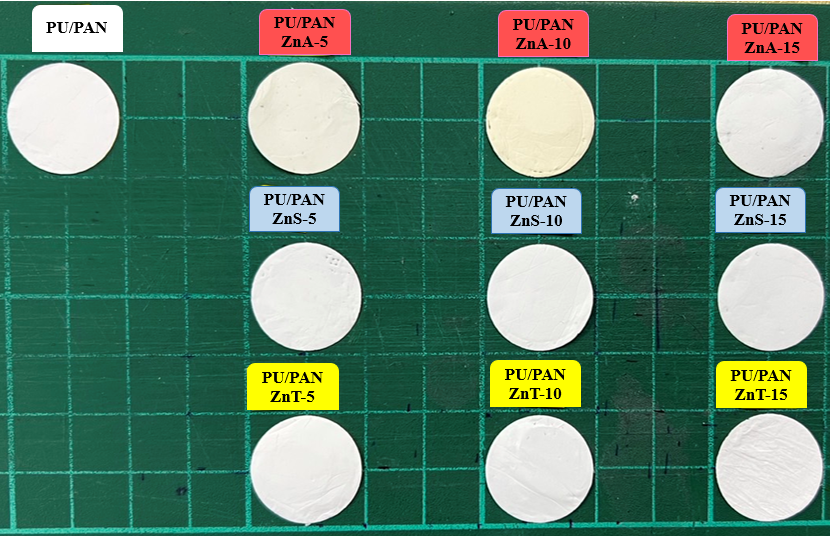

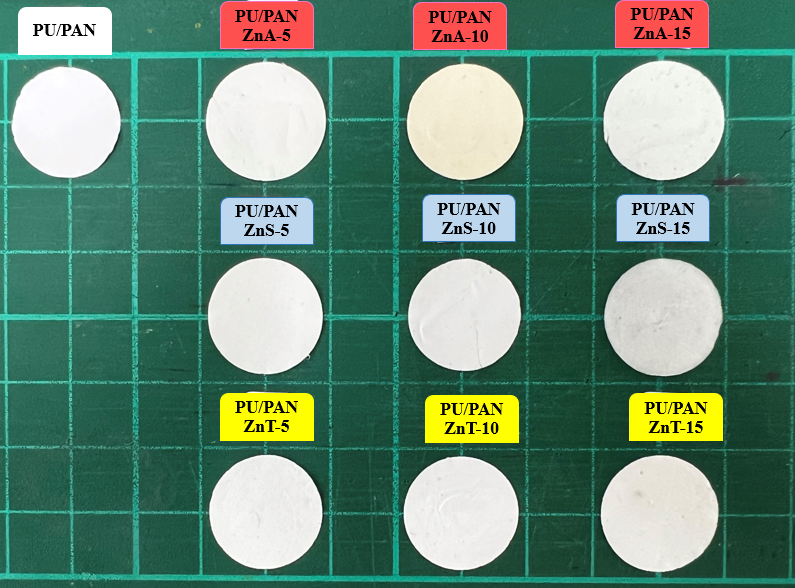


**a.**

**b.**

**Figure S5**. Representative dimensional stabilities investigation appearances of high porous polymer electrolyte after heat treatment at (a) 80^o^C, (b) 120^o^C.


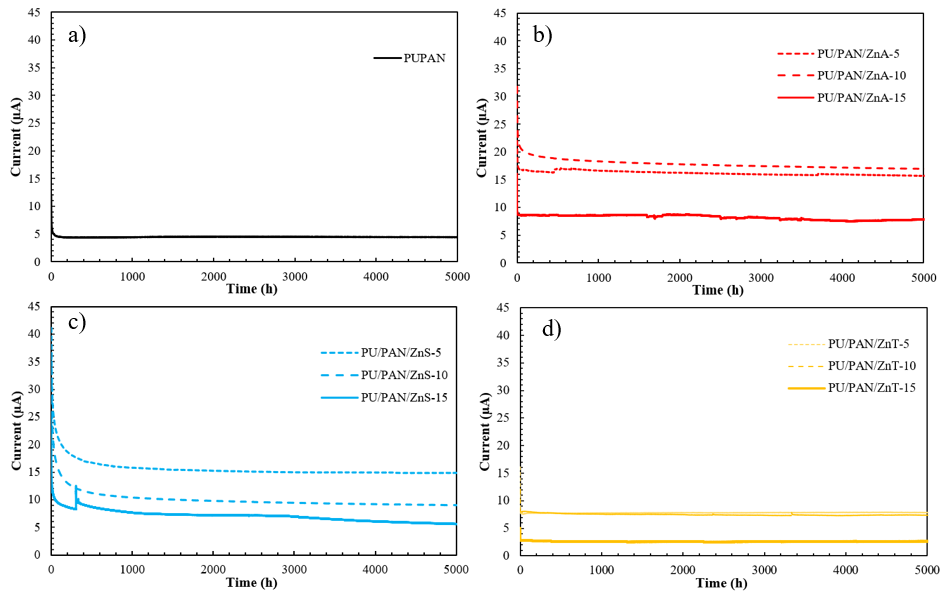


**Figure S6.** Representative chronoamperometry profiles of (a) PU/PAN, (b) PU/PAN/ZnAs, (c) PU/PAN/ZnSs, and (d) PU/PAN/ZnTs.


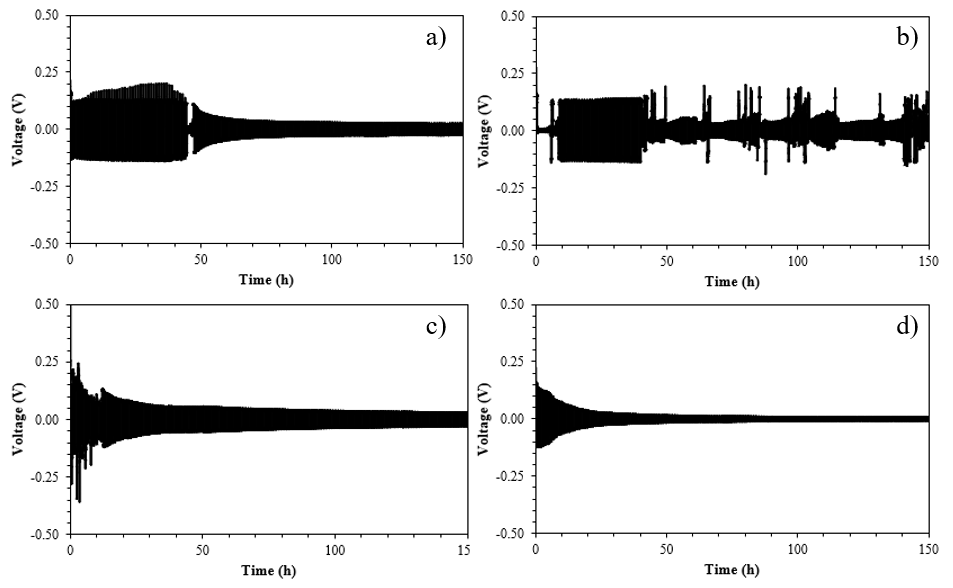


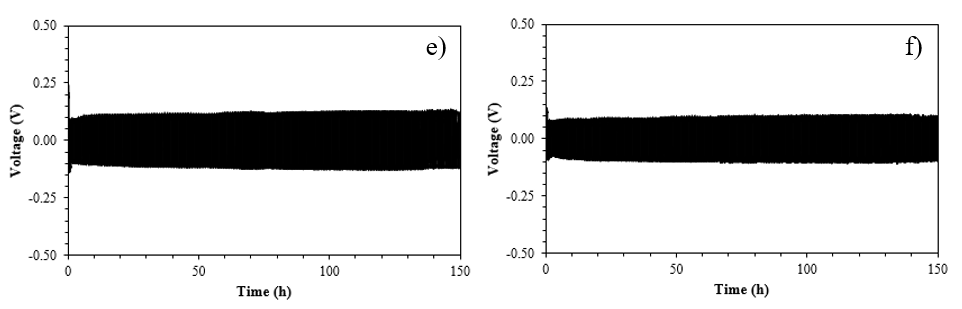


**Figure S7.** Plot of voltage profile of a) PU/PAN/ZnA-5, b) PU/PAN/ZnA-10, c) PU/PAN/ZnS-5, d) PU/PAN/ZnS-10, e) PU/PAN/ZnT-5, and f) PU/PAN/ZnT-10**.**


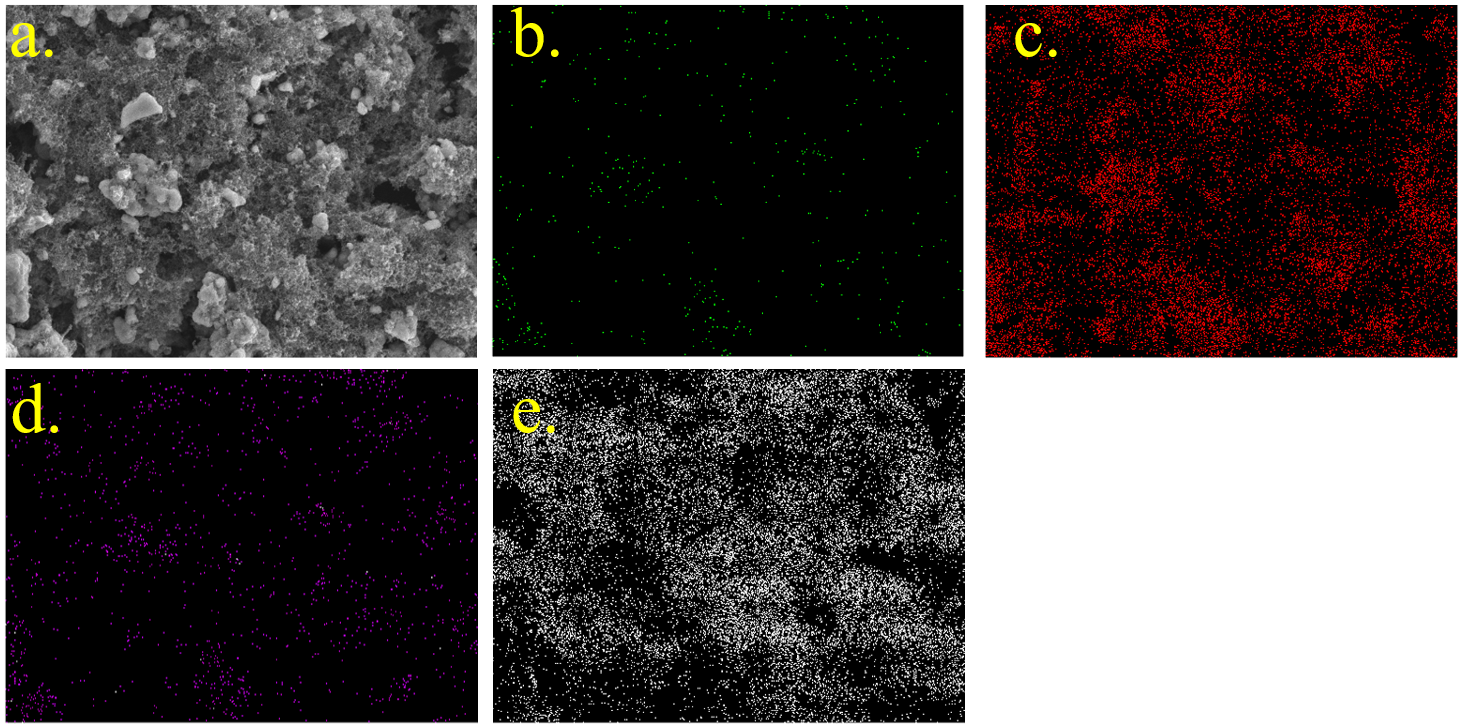


**Figure S8.** Representative of SEM elemental mapping of NVO on graphite paper cathode: a) SEM morphology, b) N mapping, c) V mapping, d) O mapping, and e) C mapping.


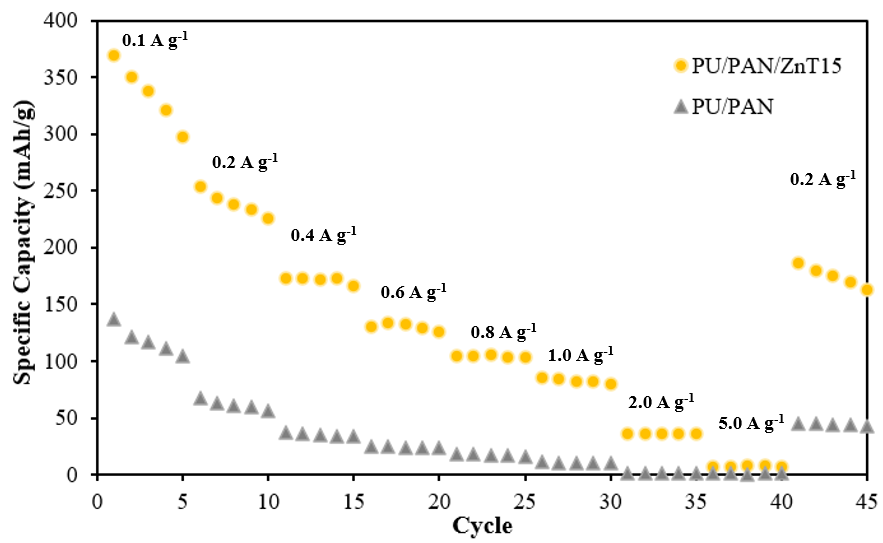


**Figure S9.** Rate performance of full cell NVO//Zn assembling with polymer electrolyte: ⚫ PU/PAN/ZnT15, and ▲ PU/PAN.

**References**

1 J. Cao, et al., Oxygen defect enriched (NH_4_)_2_V_10_O_25_⋅8H_2_O nanosheets for superior

aqueous zinc-ion batteries. *Nano Energy.* 84 (2021), 105876. 10.1016/j.nanoen.2021.105876
